# Antiviral, Antibacterial, Antifungal, and Antiparasitic Properties of Propolis: A Review

**DOI:** 10.3390/foods10061360

**Published:** 2021-06-11

**Authors:** Felix Zulhendri, Kavita Chandrasekaran, Magdalena Kowacz, Munir Ravalia, Krishna Kripal, James Fearnley, Conrad O. Perera

**Affiliations:** 1 Kebun Efi, Kabanjahe, North Sumatra 2217, Indonesia; 2 Peerzadiguda, Uppal, Hyderabad 500039, Telangana, India; dr.ckavita@gmail.com; 3 Institute of Animal Reproduction and Food Research, Polish Academy of Sciences, Tuwima 10 St., 10-748 Olsztyn, Poland; dudek06@wp.pl or; 4 The Royal London Hospital, Whitechapel Rd, Whitechapel, London E1 1FR, UK; munirrav@yahoo.co.uk; 5 Rajarajeswari Dental College & Hospital, No.14, Ramohalli Cross, Mysore Road, Kumbalgodu, Bengaluru 560074, Karnataka, India; kripalkrishna@yahoo.com; 6 Apiceutical Research Centre, Unit 3b Enterprise Way, Whitby, North Yorkshire YO18 7NA, UK; james.fearnley@beearc.com; 7 Food Science Program, School of Chemical Sciences, University of Auckland, 23 Symonds Street, Auckland CBD, Auckland 1010, New Zealand

**Keywords:** propolis, antiviral, antibacterial, antifungal, antiparasitic, phytochemical, apiculture, antioxidant, anti-inflammatory

## Abstract

Propolis is a complex phytocompound made from resinous and balsamic material harvested by bees from flowers, branches, pollen, and tree exudates. Humans have used propolis therapeutically for centuries. The aim of this article is to provide comprehensive review of the antiviral, antibacterial, antifungal, and antiparasitic properties of propolis. The mechanisms of action of propolis are discussed. There are two distinct impacts with regards to antimicrobial and anti-parasitic properties of propolis, on the pathogens and on the host. With regards to the pathogens, propolis acts by disrupting the ability of the pathogens to invade the host cells by forming a physical barrier and inhibiting enzymes and proteins needed for invasion into the host cells. Propolis also inhibits the replication process of the pathogens. Moreover, propolis inhibits the metabolic processes of the pathogens by disrupting cellular organelles and components responsible for energy production. With regard to the host, propolis functions as an immunomodulator. It upregulates the innate immunity and modulates the inflammatory signaling pathways. Propolis also helps maintain the host’s cellular antioxidant status. More importantly, a small number of human clinical trials have demonstrated the efficacy and the safety of propolis as an adjuvant therapy for pathogenic infections.

## 1. Introduction

The present viral pandemic and the threat of antibiotic resistant bacteria illustrate the ever-increasing need to find novel pharmaceutical compounds to combat microbial pathogens. Nature-derived compounds with a myriad of pharmacological properties could hold the key to overcome the never-ending and inevitable threats. Natural products have been used as medicine to treat human diseases caused by pathogens for centuries. For example, quinine the antimalarial drug derived from Cinchona tree, long used by the indigenous South American native population and discovered by the West in the 17th century [1]. Other notable examples are artemisinin, the antimalarial drug derived from the plant *Artemisia annua*, which is an herbal plant in Chinese traditional medicine; and penicillin, a natural antibiotic derived from *Penicillium* molds [2,3]. The latter two resulted in Nobel prizes, highlighting the significance of natural product research.

Beehive-derived products such as propolis have shown tremendous potential. Propolis is plant resin collected by the bees to serve various critical functions; to provide physical protection, maintain hive homeostasis, act as an antimicrobial and immune-modulator substance, induce detoxification process, and stabilize beneficial microbiome [4,5,6,7,8,9]. Propolis has a wide range of therapeutic and health benefits for humans acting as anantibacterial, antiviral, anti-inflammatory, antioxidant, and antiproliferative agent [10,11,12,13,14,15,16]. Humans have used propolis for centuries to alleviate many ailments including pathogenic infections [17].

The present review article focuses on the antiviral, antibacterial, antifungal, and antiparasitic properties of propolis. The term ‘propolis’in this review study includes propolis from all propolis-producing bees, namely European honey bees (*Apis mellifera*), Asian honey bees (*Apis cerana*), and stingless bees of the genera *Trigona*, *Melipona*, *Geniotrigona*, *Heterotrigona*, and *Tetragonula*.

## 2. Bioactive Compounds in Propolis

Propolis comprises of wax, resin, balsam, essential oils, pollen, and plant primary and secondary metabolites—such as amino acids, minerals, vitamins, phenolics, terpenoids, tannins, and alkaloids [18,19,20,21]. The precise composition of propolis varies depending on the geographical locations, plant sources, and/or bee species [22]. Majority of propolis research in terms of its health benefits has been linked to its phenolic content [23,24,25]. Phenolic compounds are one of the largest groups of plant secondary metabolites. It is estimated that 2% of all carbon fixed by photosynthesis is converted into phenolic compounds. Even though phenolics are considered as secondary metabolites as they are not directly involved in anabolic and catabolic processes, plant phenolic compounds are paramount for plant survival as they are implicated in many essential functions such as defense mechanisms (against pathogens, insects, oxidation, and UV radiation), plant developmental signaling, and so on [26,27,28]. In this review, the terms ‘phenolic compounds’, ‘phenolics’, and ‘polyphenols’confer the same meaning and are used interchangeably, unless specified otherwise.

Quideau et al. (2011) proposed that plant phenolics should be confined to the secondary metabolites produced by shikimate/phenylpropanoid pathway or acetate/malonate pathway, or combination of both [29]. These compounds include simple phenols and polyphenols. As a general description, polyphenols consist of two phenyl rings and one or more hydroxyl substituents and their functional derivatives; such as esters and glycosides. Plant phenolics can be categorized into two major groups; flavonoids and non-flavonoids. Flavonoids share a structure of diphenyl propanes (C_6_-C_3_-C_6_), where the phenolic rings are most commonly linked by a heterocylic ring. Flavonoids and their conjugates are a major group of natural products in which over 8000 flavonoids have been identified. Some examples of flavonoids are flavanol, flavanone, pinocembrin, quercetin, gallangin, chrysin, and kaempferol [30,31].

Non-flavonoid phenolics consist of groups of compounds such as simple phenols, benzoquinones, phenolic acids, stilbenes, and lignans. Examples of simple phenols (C_6_) are catechol, resorcinol, and phloroglucinol. Benzoquinones (C_6_) include *p*-benzoquione and *o*-benzoquinone. Phenolic acids (C_6_-C_1_, C_6_-C_2_, C_6_-C_3_) can be divided into two major groups, namely benzoic acid and cinnamic acid derivatives. Phenolic acids rarely occur in free from. They are usually conjugated with sugars or other organic acids. Phenolic acids are usually part of complex structures such as lignins and hydrolyzable tannins. Examples of benzoic acid derivates are gallic acid, *p*-hydroxybenzoic acid, protocatechuic acid, syringic acid, and vanillic acid, whereas cinnamic acid derivatives are caffeic acid, ferulic acid, *p*-coumaric acid, and sinapic or sinapinic acid [27,29,30,31].

Moreover, stilbenes (C_6_-C_2_-C_6_), a relatively small group of phenolic compounds, are characterized by two phenyl moieties linked by a two-carbon methylene group. Examples of stilbenes are resveratrol and 1,2-diarylethenes. Another non-flavonoid phenolic group is lignans which consist two propylbenzene units (C_6_-C_3_) linked by the central carbon (C8) of the side chains. The C9 and C9′ positions of lignans are usually substituted with various different patterns, consequently lignans are classified into eight subgroups, namely furofuran, furan, dibenzylbutane, dibenzylbutyrolactone, aryltetralin, arylnaphthalene, and dibenzocyclooctadiene [27,29,30,31,32,33,34,35].

The phenolic constituents of propolis vary depending on the plants from which the bees collect the resin from. The common phenolics found in propolis were shown to be a combination of both flavonoids and non-flavonoid phenolics. Bankova et al. (2002) found that propolis from various regions of Europe (Bulgaria, Italy, and Switzerland) contained phenolic acids, phenolic acid esters, and flavonoids, with compounds such as pinocembrin, pinobanksin and its 3-O-acetate, chrysin, galangin, phenethyl esters of caffeic and ferulic acids being the highest in concentration [36]. In addition, Popova et al. (2017) identified a myriad of phenolics in the propolis from Poland; 13 phenolic acids such as benzoic acid, *p*-coumaric acid, and ferullic acid; 28 phenolic esters such as butyl *p*-coumarate, pentyl *p*-coumarate, and pentenyl *p*-coumarate; 30 flavonoids such as pinocembrin, pinobanksin, chrysin, galangin, and kaempferol. Kasiotis et al. (2017) investigated the composition of Greek propolis from eightdifferent regions. They found substantial amount of pinocembrin and chrysin; 361–13,992 µg/g (dry extract) and 170–9940 µg/g (dry extract), respectively [37]. However, the concentrations for other phenolics such as apigenin, galangin, pinobanksin, gallic acid, and so on, ranged from ‘undetected’to 2529 µg/g (dry extract) [37].

Shi et al. (2012) found caffeic acid, *p*-coumaric acid, ferulic acid, isoferulic acid, 3,4-dimethylcaffeic acid, pinobanksin, chrysin, pinocembrin, galangin, pinobanksin-3-acetate, and caffeic acid phenethyl ester were the dominant phenolics in propolis from various provinces of China, covering geographically diverse regions [38]. In addition, Chen et al. identified and isolated several prenylflavanones; propolin A-F as bioactive phenolic components of propolis collected from various regions of Taiwan [39,40]. Interestingly, they demonstrated that seasons, rather than geographical locations, played a major role in determining the total phenolics composition of Taiwanese propolis [41]. Trusheva et al. (2011) identified alk(en)ylresorcinols and propolin C, D, F, and G as phenolic components of propolis from a region in Indonesia [42]. Furthermore, Kasote et al. (2017) identified gallic acid, naringin, caffeic acid, *p*-coumaric acid, ferulic acid, quercetin, cinnamic acid, kaempferol, chrysin, galanginin, and caffeic acid phenethyl ester as main phenolic components of propolis collected from various regions in India [43].

Arguably, the most extensive propolis studied is Brazilian propolis. Brazilian propolis is usually categorized into three types based on its physical appearance: green, red, and brown propolis. The botanical source of Brazilian green propolis is *Baccharis dracunculifolia* [44]. The main phenolic compound is typically artepillin C, in addition to moderate concentration of flavonoids such as kaempferol and kaempferide. Brazilian green propolis also contains phenolic acids such as *p*-coumaric acid and 3-prenyl-4-hydroxycinnamic acid [19,44,45,46]. In addition, Brazilian red propolis contains phenolics such as retusapurpurin A and B, formononetin, biochanin A, vestitol, neovestitol, and daidzein [47,48]. The botanical source of Brazilian red propolis is purportedly *Dalbergiaecastophyllum* [47,49,50]. Furthermore, the main phenolic compounds in Brazilian brown propolis were shown to be galangin, pinocembrin, chrysin, apigenin, pinobanksin, and apigenin [51]. To date, the botanical origin of brown propolis has not been determined; it is most likely diverse in botanical sources [52]. Table 1 illustrates the profile of phenolic compounds of propolis from different sources.

Another important group of bioactive compounds of propolis is terpenoids. Terpenoids are secondary plant metabolites that play crucial roles in various plant functions, such as parts of hormone-mediated signaling and electron transfer systems, antioxidants, and plant defense mechanisms against insects and pathogens [68]. The synthesis of terpenoids in plants starts from the conversion of the 5-carbonisoprenoid precursors into various structurally distinct terpenoid core scaffolds which are then structurally modified further into >50,000 terpenoids. The enzymatic modification processes of core scaffolds to various terpenoids are catalyzed by terpene synthases and cytochrome P450 monooxygenase (P450) enzymes [69,70,71].

Bankova et al. (2002) found propolis samples from Sicily had very little phenolic compounds but instead contained diterpenic acids as the main bioactive compounds [36]. Melliou et al. (2007) investigated volatile compounds of propolis from various regions of Greece [72]. They found that the predominant volatiles are terpenoids, withα-pinene, junipene, and δ-cadinene being some of the predominant compounds [72]. In addition, Popova et al. (2010) identified at least 37 diterpenes from propolis from various regions of Greece, with isocupressic acid, pimaric acid, communic acid, and 14,15-dinor-13-oxo-8(17)-labden-19-oic acid being the most dominant terpenoids [73]. The same group identified 32 diterpenes isolated from various propolis samples from Malta [74]. Two specific diterpenes; daucane diterpene esters of hydroxybenzoic acids, were isolated and linked to botanical source *Ferula communis*. They also demonstrated that all samples had high antibacterial activity against *Staphylococcus aureus*, however only propolis samples with high concentration of terpenyl esters were shown to have high antifungal activity against *Candida albicans* [74].

Stingless bees from the tropics appear to have terpenoids as the predominant bioactive compounds. Zhao et al. (2017) identified at least 28 bioactive compounds (phenolic acids, flavones, terpenoids, and phytosterol) extracted from *Heterotrigona itama* propolis from Malaysia with two terpenoids; 24(*E*)-cycloart-24-ene-26-ol-3-one and 20-hydroxy-24-dammaren-3-one being the predominant compounds [56]. In addition to phenolics, Nazir et al. (2018) found that propolis extracted from stingless bees *Geniotrigona thoracica* in the Malaysian region of Kota Bharu, Kelantan contained various terpenoids such as fren-9(11)-en-2-alpha-ol, lup-20(29)-ene-3,21-dione, 28-hydroxy-, and beta-amyrenol [55]. Pujirahayu et al. (2019) identified various cycloartane-type triterpenes such as mangiferolic acid, cycloartenol, and ambolic acid from ethanolic extract of propolis from stingless bees *Tetragonula sapiens* in Sulawesi, Indonesia [75]. These terpenoids were associated with its propolis botanical source which was *Mangifera indica* [75]. Furthermore, Iqbal et al. (2019) found that some propolis samples from stingless bees appeared to have unusually modified terpenoids which had strong anti-angiogenic properties [76]. Health properties of terpenoids are relatively less studied compared to phenolics. However, there is growing body of evidence that propolis terpenoids have strong therapeutic benefit. Terpenoids have been shown to have anticancer, antibacterial, antiviral, antioxidant, and anti-inflammatory properties [13,76,77,78,79,80]. Further research is needed to investigate the therapeutic properties of propolis from the tropical stingless bees which appear to contain unique terpenoid compositions. Table 2 illustrates the profile of terpenoids from different sources.

Propolis also contains other compounds such as tannins, alkaloids, vitamins, amino acids, minerals, and fatty acids, albeit in small concentration [21,65,83,84,85,86]. Bioactive composition of propolis described and listed in this review is by no means exhaustive. The description should be considered and treated as examples.

**Figure 1:**
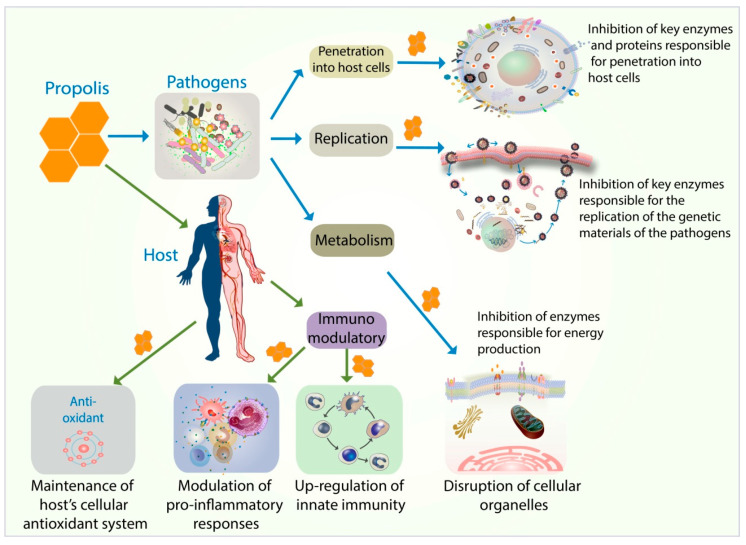
There are two distinct impacts with regards to antimicrobial and anti-parasitic properties of propolis; on the pathogens and on the host. With regards to the pathogens, propolis acts by inhibiting the ability of the pathogens to invade the host cells (by forming a physical barrier and inhibiting enzymes and proteins needed for invasion into the host cells). In addition, propolis inhibits the replication process of the pathogens by inhibiting the enzymes needed for the replication of the pathogens’ genetic materials. Propolis also inhibits the metabolic processes of the pathogens by disrupting cellular organelles and components responsible for energy production. With regard to the host, propolis acts as an immunomodulator. It upregulates the innate immunity and modulates the inflammatory signaling pathways. Propolis also helps maintain the host’s cellular antioxidant status throughout the infection.

Antimicrobial and antiparasitic properties of propolis should be considered at two levels, its impact on the pathogen itself and the impact on the host. With respect to the latter, propolis has well-established immunomodulatory effects [87,88]. Figure 1 summarizes various mechanisms of action of propolis in exerting its antiviral, antiviral, antifungal, and antiparasitic effect. Propolis and its bioactive components exert antiviral activity through various mechanisms of action.

## 3. Antiviral Properties of Propolis

In the present review article, the antiviral properties of propolis discussed would be focused on pathogenic human viruses. Propolis has been demonstrated to have antiviral properties against a wide range of viruses. One of the earliest studies was carried out by Debiaggi et al. (1990) which investigated the propolis-derived flavonoids, namely chrysin, kaempferol, acacetin, galangin, and quercetin against various strains of herpesvirus, adenovirus, rotavirus, and coronavirus [89]. More importantly, propolis has been shown to have antiviral activity against SARS-CoV-2. Refaat et al. (2021) demonstrated that propolis delivered in a liposomal encapsulation was as effective as remdesivir in neutralizing SARS-CoV-2 in vitro [90]. Many computational and molecular docking studies suggest the efficacy of propolis and its phenolic components in interfering with many important proteins of the SARS-CoV-2, including proteases and the spike protein [91,92,93,94].

In addition, propolis is efficacious against influenza viruses. Serkedjieva et al. (1992) demonstrated that propolis-derived phenolics, especially, isopentyl ferulate exhibited strong antiviral activity against H3N2 influenza A virus [95]. Shimizu et al. (2008) investigated the antiviral efficacy of thirteen ethanolic extracts of Brazilian propolis against influenza virus A/PR/8/34 (H1N1) [96]. It was found that all extracts had antiviral properties with various level of efficacy. One extract (AF-08), in particular, was effective in reducing weight loss and prolonging the life of infected mice. Ten mg·kg^−1^ AF-08 extract was also shown to be almost as efficacious as 1 mg·kg^−1^ oseltamivir (antiviral drug) in reducing the viral load in the bronchoalveolar lavage fluids of the lungs of the infected mice [96].

Kai et al. (2014) showed that propolis-derived phenolics; apigenin, kaempferol, and coumaric acid were effective against Influenza A/PR/8/34(H1N1) and both oseltamivir- and peramivir-sensitive and resistant strains of influenza A/Toyama/26/2011 (H1N1) viruses. In addition, kaempferol was shown to reduce the viral load in the bronchoalveolar lavage fluids and prolong the survival time of the infected mice [97]. Moreover, Kuwata et al. (2011) demonstrated that water extract of propolis had antiviral activity against influenza virus A/WSN/33 (H1N1). It appeared caffeoylquinic acids were the active components that exerted the antiviral properties [98]. The same group also isolated and demonstrated that 3,4-dicaffeoylquinic acid as the anti-influenza compound in the propolis extract [99].

Propolis has been shown to have anti-HIV activity. Moronic acid, a triterpenoid, isolated from Brazilian propolis was shown to inhibit HIV activity in H9 lymphocytes [100]. In addition, Gekker et al. (2005) demonstrated that propolis extracts from various sources and regions, namely Minnesota (USA), Brazil, and China all inhibited the HIV-1 infected CD4+ lymphocyte and microglial cell cultures [101]. More importantly, propolis did not antagonize the activity of antiretroviral drugs such as zidovudine and indinavir [101]. Furthermore, Silva et al. (2019) reported that ethyl acetate extract of propolis from Ceará state (northeast Brazil) exhibited anti-HIV activity [102]. It was found that the propolis-derived phenolics—naringenin, quercetin, and diprenylcinnamic acid—were the compounds linked to the antiviral activity of propolis [102].

The most extensive research on antiviral properties of propolis has arguably been carried out in herpes viruses. Amoros et al. (1992) demonstrated that galangin, kaempferol, and quercetin had anti-herpetic activity. They also demonstrated that there were synergistic relationships among the propolis-derived phenolics which partly explained the higher activity propolis when compared to its individual components [103]. Schnitzler et al. (2010) also demonstrated the synergistic nature of the propolis-derived compounds. It was shown that propolis aqueous and ethanolic extracts had superior anti-herpetic activity when compared to the individual components such as caffeic acid, *p*-coumaric acid, benzoic acid, galangin, pinocembrin, and chrysin [104]. In addition, Bankova et al. (2015) showed that poplar propolis containing various phenolics—such as benzoic acid, *p*-coumaric acid, benzyl *p*-coumarate, benzyl ferulate, pinocembrin, and pinocembrin chalcone—exhibited antiviral activity against herpes simplex virus types 1 and 2 (HSV-1 and 2) [105].

Propolis from stingless bees also has anti-herpetic activity. Coelho et al. (2015) showed that hydromethanolic extract of stingless bees *Scaptotrigona trigona*, which contained pyrrolizidine alkaloids and C-glycosyl flavones as the active ingredients, exhibited antiviral activity against HSV-1 [106]. Moreover, propolis from *Melipona quadrifasciata* was shown to inhibit HSV-1 activity. The dichloromethane, butanol, and ethyl acetate fractions in particular showed the strongest anti-herpetic activity [107].

Anti-herpetic activity of propolis extract has also been shown in studies involving animal models. Kurokawa et al. (2011) investigated several propolis extracts in mice and demonstrated that various extracts appeared to inhibit the herpes virus at different stages of infection [108]. Various ethanolic extracts of propolis significantly reduced the viral load in brains and skins of mice infected with HSV-1 [108]. Sartori et al. (2012) demonstrated that hydroalcoholic extract (70% ethanol) of propolis reduced the severity of extravaginal lesions and histological damage in the vaginal tissue of animals infected with HSV-2 [109]. Furthermore, antiviral properties of propolis extend to various viruses such as rhinovirus, dengue virus, polio virus, rubella virus, picornavirus, and measles virus [110,111,112].

### Mechanisms of Action of Antiviral Properties of Propolis

Molecular docking and in silico studies unveiled the potential mechanisms of action employed by propolis and its components in inactivating SARS-Cov-2. Refaat et al. demonstrated that rutin and caffeic acid phenethyl ester inhibited both 3CL-protease and S1 spike protein of SARS-Cov-2 [15]. Caffeic acid phenethyl ester was also shown to interfere with the highly conserved residues (substrate-binding pocket) of M^pro^ protein of SARS-Cov-2 [113]. In addition, molecular docking studies by Sahlan et al. (2021) and Dewi et al. (2021) demonstrated that Sulabiroins A, (2S)-5,7-dihydroxy-4′-methoxy-8-prenylflavanone acid, glyasperin A, and broussoflavonol F (propolis-derived compounds) could potentially bind to various residues of M^pro^ catalytic sites and consequently inhibit the activity of the M^pro^ protein of SARS-Cov-2 [93,94].

Kwon et al. (2020) demonstrated that kaempferol and *p*-coumaric acid prevented the entry of human rhinovirus and also inhibited the viral replication in HeLa cells [112]. In addition, ferulic acid isolated from propolis was demonstrated to inhibit the activity of porcine parvovirus [114]. Ferulic acid inhibited and reversed the parvovirus-induced expression of pro-apoptotic genes Bid, Bad, Bim, and Bak. The expression of these genes has been shown to be associated with mitochondrial disruption and apoptosis of the host cells [114]. Propolis was also shown to enhance the expression of myxovirus resistance 1 (MX1) gene [115]. Mx proteins are the “gatekeepers” of the host cells in overcoming RNA viruses and other virus families that replicate in the host nucleus [116]. Polyphenols also help transport Zn cations into across the plasma membrane independently of plasma membrane zinc transport proteins [117]. Zn cations have been shown to inhibit the activity of viral RNA-dependent RNA polymerase [118].

Moreover, the immunomodulatory properties of propolis in affecting host immune functions were evident in the virus infection models. Propolis constituent 3,4-dicaffeoylquinic acid (3,4-diCQA) was shown to increase the expression of tumor necrosis factor-related apoptosis-inducing ligand (TRAIL) which expedited viral clearance [99]. Propolis also reduced the oxidative stress in the infected host cells by inhibiting the expression of reactive species, tyrosine nitration, and myeloperoxidase activity. Propolis also maintained the expression of catalase, an important enzyme in the cellular antioxidant system, in infected cells [109]. Additionally, propolis also induced the production of interferon-γ (IFN-γ) in HSV infection models. IFN-γ is a significant stimulator of lymphocyte migration into skin and consequently important in alleviating the symptoms of viral infections such HSV infections [108]. Table 3 summarizes and illustrates the antiviral properties of propolis and/or propolis-derived compounds.

## 4. Antibacterial Properties of Propolis

Antibacterial properties of propolis are very well documented in the scientific literature. Przybyłek and Karpiński (2019) recently reviewed the analyses of the reported data on the influence of propolis on about 600 strains of bacteria, both aerobic and anaerobic [120]. Information of particular bacterial species susceptible to propolis action as well as values of the minimal inhibitory concentration (a minimum concentration at which no microorganism growth can be observed in the assays) can be found in that review [120]. Generally, it has been shown in multiple studies that propolis exhibits more powerful antimicrobial activity against Gram-positive than Gram-negative bacteria [120,121,122]. The difference was thought to be due to the presence of bacterial hydrolytic enzymes in the outer membrane of Gram-negative bacteria, which could potentially compromise and reduce the efficacy of the active components of propolis [123,124].

It is common practice to relate the potential antimicrobial properties of propolis to its phenolic and flavonoid content. Nevertheless, Bridi et al. (2015) showed that the concentration of those components does not always correlate with observed antimicrobial activity in vitro [125]. Therefore, it has been suggested that other tests should be used to set some standards for evaluation of propolis biological activity. In fact, the plethora of active ingredients in varying combinations/concentrations is the property of propolis that can prevent bacterial resistance from occurring [126].

In addition, geographical origin appears to affect the composition of propolis which consequently affects its antibacterial properties [127,128]. It was found in particular, that propolis from the Middle East exhibits highest activity against both Gram-postive and Gram-negative strains, while that from Germany, Ireland, and Korea has the lowest activity [120]. Apart from having direct antimicrobial effect, propolis also acts synergistically with conventional antibiotics enhancing their efficacy as well as with other natural products such as honey [129,130,131].

### Mechanisms of Action of Antibacterial Properties of Propolis

With regards to its antibacterial mode of action, propolis can interfere with their pathogenic potential by increasing permeability of the bacterial cell membrane, inhibiting ATP production, decreasing bacterial mobility, disturbing membrane potential, and impairing bacterial RNA and DNA production [120,121]. Because of the complex nature of propolis in terms of its composition, it is not possible to precisely elucidate specific mechanism responsible for each of its many effects. The studies usually concentrate on some selected components or their mixtures and try to relate observed outcomes to that induced by unfractionated propolis extracts. There are many possible biochemical mechanisms that can underlie the antibacterial actions of propolis. They have been covered in the extensive literature and also summarized in recent reviews [120,121,122]. Therefore, in this article, we will focus on the studies that represent some novel aspects in the field.

It has been shown in a recent study that apart from biochemical pathways, there is also physical mechanism that can contribute to the biological activity of propolis [132]. Namely, propolis deposited on a surface was found to generate a layer of water which effectively excludes colloidal particles (termed exclusion zone (EZ) water). The phenomenon is based on the electrokinetic process, depended on the presence of negatively charged functional groups characteristic to many chemical components of the propolis [132,133]. Therefore, the colloid-excluding property has very generic character, largely independent of the exact composition and origin of the propolis. From the physical perspective, all bacteria or viruses suspended in aqueous solution (e.g., our body fluids or mucosal lining) are colloids. Thus, it has been proposed that propolis can prevent pathogens from accessing the surface (e.g., respiratory epithelium) by creating the physical barrier in the form of EZ [132].

There are few recent studies showing that propolis-functionalized textiles acquire antibacterial properties and can be potentially used in medical field, e.g., as wound dressing [134,135,136]. In one case, propolis was chemically bonded to the cotton fibers and resisted several washing cycles [134]. Bacteria-free zone could be observed next to such propolis-functionalized textile in so called disk diffusion method. Any diffusion of bounded propolis components could hardly take place, yet EZ could be created as this mechanism does not require chemical (contact) interaction with bacteria. Other EZ-generating materials were also suggested to potentially provide first line of defense against microorganisms colonizing surfaces (e.g., in healthcare facilities) [137].

Considering further physicochemical characteristics of propolis, the presence of fixed negative charges in its chemical constituents goes in hand with mobile protons. Protons (positive charges) are diffusible and providing propolis its acidic pH [138]. It is recognized that cationic agents are able to reduce negative charges of bacterial cells, promote membrane permeability, and consequently induce bacterial cell death [139]. Therefore, higher bactericidal activity of propolis against Gram-positive compared to the Gram-negative bacteria could be also related to the less negative surface charge of Gram-positive strains and its higher susceptibility to mobile protons. Such a link is not yet confirmed but would deserve further studies and could possibly help discriminate, screen, and isolate propolis samples that have higher bactericidal activity against Gram-negative species.

## 5. Antifungal Properties of Propolis

Antifungal activity of propolis has been well documented in the literature. It is known to be influenced by the variation in chemical composition of propolis [140]. This variation in antifungal effect has been reported in numerous studies analyzing the effect of propolis from different geographic origin against different fungal species, particularly of clinical interest [141,142,143,144,145]. Propolis is known to possess antifungal activity against fungal species such as *C. albicans*, *C.parapsilosis*, *C. tropicalis*, *C. glabrata* [131,146]. It has shown an aflatoxigenic property against fungi like *Aspergillus flavus*, where it inhibited conidial growth of the fungi [147]. In this review article, focus has been laid on the fungal species affecting humans and related clinical studies.

Propolis extract has displayed excellent performance regarding in vitro tests performed against yeasts identified as onychomycosis agents. In these experiments, it was observed that in low concentrations, propolis acts as a fungistatic and fungicidal agent. Ota et al. (2001) carried out experimental studies on Brazilian propolis activity against 80 strains of *Candida* yeast (20 strains of *C. albicans*, 20 strains of *C. tropicalis*, 20 strains of *C. krusei*, and 15 strains of *C. guilliermondii*) [141]. A clear antifungal activity of propolis was reported in the following order of sensitivity: *C. albicans* > *C. tropicalis* > *C. krusei* > *C. guilliermondii*, with *C. albicans* being the most sensitive and *C.guilliermondii* being the most resistant. The minimal inhibitory concentrations (MICs) were in the range of 8–12 mg/mL. A reduction in the number of *Candida* species in saliva was also observed in patients with full dentures who used a hydroalcoholic extract of propolis [141].

Both green and red Brazilian propolis have displayed antifungal activity against different fungal species of *Trichophyton*, which cause dermatophytosis, with red propolis being more efficacious [143]. In addition, n-hexane extract of Brazilian red propolis has efficacy against *Candida* spp. resistant to antifungal agents like fluconazole [148]. Oliveira et al. (2006) tested an alcoholic extract of Brazilian propolis against fungal isolates of *C. parapsilosis*, *C. tropicalis*, *C. albicans*, and other yeast species obtained from onychomycosis lesions. It was observed that the concentration of propolis which was capable of inhibiting all of the yeasts contained 50 μg/mLof flavonoids while yeast cell death was promoted at 20 μg/mLof flavonoids. The most sensitive species was recorded as *Trichosporon* sp. [149].

A study was conducted by Quiroga et al. (2006) to demonstrate the antifungal activity of propolis originating from the northwest of Argentina [150]. Their study focused on the antimycotic and cytotoxic activities of partially purified propolis extract on yeasts and xylophagous and phytopathogenic fungi. A comparison of propolis activity was also carried out with compounds like pinocembrin and galangin isolated from the same propolis and also with the synthetic drugs viz. ketoconazole and clotrimazole. They observed that partially purified propolis extract was capable of inhibiting fungal growth. The comparison of its relative biocide potency and cytotoxicity with the isolated compounds and synthetic drugs showed that the propolis was a reliable source of antifungal agent [150]. Another study was conducted by Agüero et al. (2010) using Argentinian propolis extract. The antifungal activity was tested against a range of fungi and yeasts. Most susceptible species were reported to be *Microsporum gypseum*, *Trichophyton mentagrophytes*, and *Trichophyton rubrum*. All the other dermatophytes and yeasts tested were strongly inhibited by different propolis extracts (MIC values being between 16 and 125 μg/mL) [151].

Falcao et al. (2014) carried out a study on antifungal activity of Portuguese propolis and its potential floral sources against *C.albicans*, *T.rubrum*, and *Aspergillus fumigatus.* A significant effect was observed with *T. rubrum* and least effect was showed on *A.fumigatus* [152]. Szweda et al. (2010) carried out an in vitro analysis of ethanolic extract of propolis (Poland), essential oils and silver nanoparticles dropped on TiO_2_ for their antifungal activity against fluconazole-resistant *C.albicans*, *C.glabrata*, and *C.krusei.* They observed a satisfactory fungicidal activity of all the samples against *C.albicans* and *C.glabrata* isolates thus representing high potential to control and prevent candidiasis [153]. Boisard et al. (2015) assessed the antifungal activity of organic extracts of French propolis against various fungi and observed effective activity against *C. albicans* and *C.glabrata* but only weak activity towards *A.fumigates* [154].

It appears delivery methods and/or vehicles could potentially affect the antifungal activity of propolis. The study conducted by Bruschi et al. (2011) evaluated the in vitro antifungal activity of propolis ethanolic extract (PEE) and propolis microparticles (PMs) obtained from a Brazilian propolis sample against clinical isolates of yeast responsible for vulvovaginal candidiasis. Their observation revealed that both PEE and PMs were efficient in inhibition of *C.albicans* and non-*C. albicans* [144]. Beretta et al. (2013) studied the fungicidal effect of propolis extracts, propolis matricial microparticles, and propolis soluble dry extract in an in vivo experimental animal model [155]. The effect was evaluated 6–8 h post treatment and against three *C. albicans* morphotypes (yeast, pseudohyphae, and hyphae). Among all the extracts, PEE was the most potent and was followed by PSDE, PM, and PWE.Bonfim et al. (2020) conducted an in vitro and in vivo study to assess efficacy of a new mucoadhesive thermoresponsive platform for propolis delivery (MTS-PRPe) in a preclinical murine model of vulvovaginal candidiasis treatment caused by *C.albicans* [156]. They carried out chemical analysis, an assessment of the rheological and mucoadhesive properties of propolis formulations, in vitro and in vivo antifungal evaluations, histological evaluations, and electron microscopy of the vaginal mucosa. The authors observed antifungal activity of propolis extract and MTS-PRPe against the standard strain and a fluconazole-resistant clinical isolate of *C.albicans*, in vitro and in vivo. They demonstrated that the MTS-PRPe did not negatively affect the efficacy of propolis [156].

### Antifungal Mechanisms of Action of Propolis

Wagh (2013) extensively reviewed studies on propolis and its pharmacological properties, and concluded that the presence of phenolic compounds in propolis was considered responsible for fungicidal activity against *C. pelliculosa*, *C. parapsilosis*, *C. famata*, *C. glabrata*, and *Pichia ohmeri* [157]. Banskota et al. (2011) reported that the constituents of propolis such as 3-acetylpinobanksin, pinobanksin-3-acetate, pinocembrin, *p*-coumaric acid, and caffeic acid out of 26 or more constituents exhibited anti-fungal activity [158]. Agüero et al. (2010) proposed from their observation that the main bioactive compounds responsible for antifungal activity in the propolis extract were found to be 2′,4′-dihydroxy-3-methoxychalcone and 2′,4′-dihydroxychalcone. Both were reportedly highly active against clinical isolates of *T. rubrum* and *T. mentagrophytes* (MICs and MFCs were recorded between 1.9 and 2.9 μg/mL). Additionally, galangin, pinocembrin, and 7-hydroxy-8-methoxyflavanone were isolated from propolis samples and *Zuccagnia punctata* exudates, which displayed moderate antifungal activity [151]. Boisard et al. (2015) carried out in vitro evaluation of antifungal and antibacterial activities of aqueous and organic extracts of a mixture of French propolis samples on human pathogenic fungi, two yeasts (*C. albicans* and *C. glabrata*) and one filamentous opportunistic mold (*A. fumigatus*). They suggested from the results obtained that high content of flavonoids was responsible for the antifungal activity of propolis against *C.albicans* and *C.glabrata* species [154].

It is proposed that the antifungal activity of propolis is mainly due to its ability to induce apoptosis through metacaspase and Ras signaling [159]. Furthermore, propolis disrupts the expression of various genes (HST7, GIN4, VPS34, HOG1, ISW2, SUV3, MDS3, HDA2, KAR3, YHB1, NUP85, CDC10, MNN9, ACE2, FKH2, and SNF5) involved in pathogenesis, cell adhesion, biofilm formation, filamentous growth, and phenotypic switching. Propolis also inhibits the transition process from yeast-like to hyphal growth [159]. Propolis, in particular its phenolic component pinocembrin, appears to disrupt several critical cellular processes in a dose-dependent manner, namely energy homeostasis and mycelia growth. Pinocembrin is shown to reduce the phosphorylated adenosine nucleotides levels in hyphae of *Penicillium italicum*. Pinocembrin also damages the structure of the hyphae and the cell membrane causing the ionic leakage and soluble protein in *P. italicum* [160]. Table 4 summarizes and illustrates the antifungal properties of propolis and/or propolis-derived compounds.

## 6. Anti-Parasitic Properties of Propolis

Propolis has been demonstrated to have anti-parasitic properties against various intracellular and extracellular pathogenic protozoa. Siheri et al. (2016) found that various extracts of propolis from different regions of Libya had anti-plasmodial activity in vitro with different level of efficacy [161]. The EC_50_ ranged from 3.4 to 53.6 µg·mL^−1^ [161]. In addition, propolis extracts from four different regions of Iran were shown to be anti-plasmodial [162]. Dichloromethane extracts appeared to have stronger activity in vitro when compared to 70% ethanol and ethyl acetate extracts. Propolis extracts extended the survival of the *Plasmodium falciparum*-infected mice. However, propolis did not prevent their mortality [162].

AlGabbani et al. (2017) investigated the effect on methanolic extract on *P.chabaudi*-infected mice [163]. The reduction of parasitemia by propolis appeared to be dose dependent and the reduction up to 70% at 100 mg·kg^−1^ propolis extract was achieved. It was also shown that propolis treatment reversed the oxidative stress associated with the infection. Interestingly, propolis treatments significantly increased interferon-γ and the inflammatory TNF-α, illustrating the immunomodulatory properties of propolis [163]. Propolis treatments were also shown to significantly improve the histological appearance of the spleens of the infected mice, with the highest concentration (100 mg·kg^−1^) of propolis almost completely reversed the spleen damage caused by the *P.chabaudi* infection [163].

Silva et al. (2017) investigated three types of Brazilian propolis; red, green, and brown against *Trypanosoma cruzi* Y strain and found that all three types had trypanocidal activity [164]. However, only the activity of red propolis persisted after 96 h [164]. In addition, Otoguro et al. (2012) investigated the effect of phenolic compounds of propolis against *Trypanosoma brucei brucei* and found that two particular caffeic acid esters; β-phenethyl caffeate, farnesyl caffeate had strong antitrypanosomal activity in-vitro [165]. It was demonstrated that β-phenethyl caffeate had 18-fold stronger activity compared to farnesyl caffeate. They postulated that the presence of β-phenethyl group was critical in the antitrypanosomal activity of the caffeic acid esters [165].

Omar et al. (2016) demonstrated that Nigerian red propolis and its individual phenolic constituents such as liquiritigenin, pinocembrin, vestitol, medicarpin, 8-prenylnaringenin, 6-prenylnaringenin, propolin D, macarangin, and dihydrobenzofuran had moderate anti-trypanosomal activity against standard drug-sensitive *T. brucei brucei* clone and two pentamidine-resistant types [166]. The same group also identified several Nigerian propolis-derived compounds; three xanthones; 1,3,7-trihydroxy-2,8-di-(3-methylbut-2-enyl)xanthone, 1,3,7-trihydroxy-4,8-di-(3-methylbut-2-enyl)xanthone, and 1,7-dihydroxy-8-(3-methylbut-2-enyl)-3-(methylbut-2-enyloxy) xanthone and three triterpenes: ambonic acid, mangiferonic acid, and a mixture of α-amyrin with mangiferonic acidhaving anti-trypanosomal activity against *T. brucei brucei* [167]. However, the individual compounds did not have superior trypanocidal activity compared to the crude extract [167].

Gressler et al. (2012) demonstrated that propolis had trypanocidal activity against *T. evansi* in-vitro [168]. All trypomastigotes were inactivated by 10 µg·mL^−1^ propolis extract in 1 h. However, its activity did not translate into in vivo. Infected rats were treated orally with propolis with increasing concentrations of 100, 200, 300, and 400mg·kg^−1^ body weight. All rats died from the infection, with the rats treated with the 200–400 mg·kg^−1^ body weight survived slightly longer compared to 100 mg·kg^−1^ [168]. Nweze et al. (2017) appeared to confirm the inefficacy of propolis extract in vivo in treating trypanosomiasis [169]. All infected rats treated died at the end of the trial. However, the rats treated with the higher concentration of propolis extract (400 and 500 mg·kg^−1^ body weight) had less severe secondary parameters such as less parasitemia, higher packed cell volume, higher hemoglobin concentrations and less weight loss [169]. Various propolis extracts from various regions such as Middle East, Europe, and South America have also been demonstrated to have potent anti-trypanocidal activity [170,171,172]. Furthermore, propolis was shown to have anti-parasitic activity against a variety of other protozoan parasites, namely *Leishmania amazonensis*, *Trichomonas vaginalis*, *Cryptosporidium* spp., *Blastocystis* spp., *Toxoplasma gondii*, and *Giardia lamblia* [170,173,174,175,176,177,178].

### Antiparasitic Mechanisms of Action of Propolis

Propolis, mainly due to its plant secondary metabolite content (phenolics and terpenoids), works against protozoan parasites through several mechanisms of action. Taxifolin-3-acetyl-4′-methyl ether (flavonol derivative) and bilobol (alkyl resorcinol) isolated from Libyan propolis appears to exert anti-trypanosomal activity by inducing cell lysis, disrupting phospholipid metabolism and depleting the pathogens of important lipids such as phosphatidyl glycerol (PG) and phosphatidyl inositol (PI) lipids [179]. In addition, rosmarinic acid and apigenin (both phenolics that are often found in propolis) induce physical damage in the form of cell lysis, cytoplasmic condensation, and kinetoplast and nuclear DNA aggregation in *L. donovani*. These propolis phenolics also promote cell arrest at the G0/G1 phase and inducediron chelation [180].

Resveratrol, a phenolic often associated with red wine but it is also present in propolis from certain region, exerts anti-trichomonal activity by affecting hydrogenosome metabolism [181,182,183]. Hydrogenosome is an organelle responsible for energy production and involved in redox balance in eukaryotes including protozoa [184]. Resveratrol also induces changes in the activity and expression of proteins associated with hydrogenosome metabolism—namely [Fe]-hydrogenase (Tvhyd), pyruvate-ferredoxin oxidoreductase, and heat shock protein 70 (Hsp70)—consequently causing hydrogenosome dysfunction and inactivation of the parasites [182]. In addition, kaempferol affects the adhesion mechanisms of the parasites by modifying the expression of actin, myosin II heavy chain and cortexillin II [185]. Epicatechin was shown to induce similar effect to resveratrol and kaempferol, such as the modification of the expression of the heat shock protein 70, myosin II heavy chain, and actin [186]. Additionally, epicatechin also affects the expression offructose-1,6-biphosphate aldolase and glyceraldehyde-phosphate dehydrogenase which are energy metabolism-related enzymes [186].

Apigenin, quercetin, and caffeic acid exert anti-parasitic effects through different mechanisms of action. Apigenin induces the inhibition of cell proliferation and upregulation of the expression of reactive oxygen species (ROS) in *L. amazonensis*. Apigenin also induces swelling in the parasitic mitochondria and consequently alters the mitochondrial membrane potential of the parasite [187]. Quercetin treatment significantly increases the production of ROS and induced mitochondrial dysfunction and membrane potential disruption in *L. amazonensis* [188]. Quercetin also appears to affect parasitic DNA synthesis by inhibiting the rate limiting ribonucleotide reductase through iron chelation. The removal of iron destabilizes tyrosyl radicals needed for the catalyzing activity of ribonucleotide reductase [189].

Moreover, caffeic acid induces morphological changes in the parasitic cells, the integrity of cellular plasma membrane and mitochondria, and consequently promoted apoptosis. Caffeic acid also appears to increase the inflammatory response of the infected macrophages by promoting the expression of ROS and TNF-α while reducing the expression of IL-10 and the availability of iron which significantly increases the anti-parasitic activity of the macrophages [190].

Anti-parasitic activity of propolis can also be attributed to its terpenoid content. Lupane, which has been identified in propolis, was shown to have anti-parasitic activity against *L. amazonensis* by inducing morphological changes such as vacuolization of cytosol, formation of lipid body and the disruption of mitochondria. Molecular docking studies also demonstrate that lupane has a strong affinity to DNA topoisomerase [21,191].

Maslinic acid and ursolic acid, pentacyclic triterpenoids identified in propolis from stingless bees *Tetragonula laeviceps* and *Tetrigona melanoleuca*, have been shown to have antiparasitic activity [192]. Maslinic acid appears to inhibit parasitic proteases including proteases in the surface protein complex crucial for invading host cells and metalloproteases [193,194]. In addition, ursolic acid acts by inhibiting glyceraldehyde-3-phosphate dehydrogenase (GAPDH), an important glycolytic enzyme in *T. brucei* [195]. The anti-parasitic activity of ursolic acid is also related to its ability to induce caspase 3/7-independent programmed cell death [196].

Furthermore, the anti-parasitic effect of limonene, α-terpineol, and 1,8-cineole (monoterpenes in propolis) is related to their ability to increase the fluidity and permeability of the plasma membrane of the parasites leading to cell lysis [197,198,199,200]. Limonene also has the ability to inhibit the development of the parasites by downregulating the isoprenylation of proteins, which is an essential modification of proteins in eukaryotic cells [201]. Moreover, linalool (another propolis monoterpene) induces morphological changes and cell lysis of the parasites. Linalool also supports macrophages in overcoming the infection by upregulating the production of nitric oxide which induces cytoxicity on the parasites [199,202]. Table 5 summarizes and illustrates the antiparasitic properties of propolis and/or propolis-derived compounds.

## 7. Human Clinical Trials

More importantly, the antimicrobial properties of propolis have been translated to human clinical trials. Silveira et al. (2021) conducted a single-center randomized placebo-controlled trial investigating the efficacy of the Brazilian green propolis extract as an adjuvant for treating hospitalized COVID-19 patients [203]. It was found that propolis was safe (no adverse event was recorded) and efficacious in reducing the length of hospital stay. The patients who were given 400 mg/day and 800 mg/day had a median of hospital stay of 6 and 7 days, respectively, compared to 12 days in the patients of the placebo arm. Furthermore, the patients given the higher dose of propolis, i.e., 800 mg/day also had less incidence of acute kidney damage associated with COVID-19 [203].

In addition, Esposito et al. (2021) demonstrated that propolis extract was efficacious in treating mild upper respiratory tract infections [204]. They investigated the efficacy of propolis in alleviating symptoms of respiratory tract infections such as sore throat, muffled dysphonia, and swelling and redness of the throat. Eighty three percent of the patients of the propolis arm recorded remission of the symptoms after three days of treatment, whereas the placebo-arm patients had at least one symptom after three days. No adverse event was recorded during the trial [204]. Other clinical trials on COVID-19 and ear, nose, and throat infections using combination treatments with propolis as one of the bioactive ingredients had also been shown to be efficacious in alleviating symptoms, and more importantly, safe [205,206,207,208].

We have covered the clinical trials of anti-herpetic properties of propolis in our previous review article [16]. Additionally, majority of the clinical trials of the antibacterial and antifungal (especially incandidiasis caused by *C. albicans*) properties of propolis were carried out in the oral health and dentistry sphere which our group covered in the same review article [16]. To our knowledge, anti-parasitic effect of propolis has not been investigated in human clinical trials.

## 8. Conclusions

Propolis is a complex phytocompound made from resinous and balsamic material harvested by bees from flowers, branches, pollen, and tree exudates.It is rich in polyphenols, especially flavonoids and phenolic acids, and has significant antiviral, antibacterial, antifungal, and antiparasitic properties shown mainly in in-vitro and in-vivostudies. Besides flavonoids, propolis also contains aromatic acids and esters, aldehydes and ketones, terpenoids and phenylpropanoids, steroids, amino acids, polysaccharides, and many other organic and inorganic compounds. However, the composition of propolis is very variable. It depends on the geographical region and the plants from which the bees extract their nectar. It is extensively consumed in various parts of the world because of its reputation as a health promoting agent including immunomodulatory, antiviral, antibacterial, antifungal, and antiparasitic properties.

Severe acute respiratory syndrome coronavirus 2 (SARS-CoV-2) promotes challenging immune and inflammatory phenomena. Recently, in controlled, randomized clinical trials, propolis has been shown to affect clinical benefits on hospitalized COVID-19 patients.Propolis-derived phenolics, especially, isopentyl ferulate exhibited strong antiviral activity against H3N2 influenza A and against influenza virus A/PR/8/34 (H1N1). Standardized propolis extracts reduced the viral load in the bronchoalveolar lavage fluids of the lungs of the infected mice. Propolis has shown inhibition of HIV activity in H9 lymphoblastoid cell lines. Propolis-derived phenolics were linked not only to anti-HIV but also to anti-herpetic activity.

Antibacterial property of propolis is well documented. It has been shown that propolis exhibit greater antimicrobial activity against Gram-positive bacteria than Gram-negative bacteria. Again, the antimicrobial property is attributed to the phenolic compounds found in propolis. Apart from biochemical pathways, there is also physical mechanism that can contribute to the antibacterial activity of propolis. It is based on the electrokinetic process and depends on the presence of negatively charged functional groups characteristic to many chemical components of propolis. When propolis deposits on a surface, it combines with a layer of water to form exclusion zones (EZ), which effectively excludes colloidal particles. Thus, it has been proposed that propolis can prevent pathogens from accessing the surface (e.g., respiratory epithelium) by creating the physical barrier in the form of EZ.

Propolis extracts have shown excellent activity against several strains of fungi and yeasts. However, it appears that the delivery methods and/or vehicles could potentially affect the antifungal activity of propolis. It is proposed that the antifungal activity of propolis is mainly due to its ability to induce apoptosis through metacaspase and RAS GTPase signaling.

Propolis has been demonstrated to have anti-parasitic properties against various intracellular and extracellular pathogenic protozoa. Propolis extracts extended the survival of the *Plasmodium falciparum*-infected mice. The reduction of parasitemia by propolis appears to be dose dependent. The mode of action was mainly due to its phenolic and terpenoid content. The phenolic compounds rosmarinic acid and apigenin that are often found in propolis induce physical damage in the form of cell lysis, cytoplasmic condensation, and kinetoplast and nuclear DNA aggregation in protozoan parasites to produce anti-parasitic activity.

Since the composition of propolis varies from region to region and from the types of bees used for propolis collection, there is a need to standardize propolis extracts for future use byhuman beings. Also, because there is a myriad of compounds in propolis, there is an urgent need to develop tests to set some standards for the evaluation of propolis biological activity.

## Figures and Tables

**Table 1:** The profile of phenolic compounds of propolis from various sources. These are for illustrative purposes only and by no means exhaustive.

Country	Extracts	Species	Chemical Composition Profile	References
**European honey bees**				
Bulgaria	hydroethanolic	*Apis mellifera*	Pinocembrin, pinobanksin, pinobanksin-3-O-acetate, chrysin, galangin, prenyl esters of caffeic acid and ferulic acid	[36,53]
Italy	hydroethanolic	*Apis mellifera*	Pinocembrin, pinobanksin-3-O-acetate, chrysin, galangin, benzyl caffeate, and caffeic acid phenethyl ester	[36]
Switzerland	hydroethanolic	*Apis mellifera*	Pinocembrin, pinobanksin-3-O-acetate, galangin, phenolic glycerides	[36]
Poland	hydroethanolic	*Apis mellifera*	Benzoic acid, *p*-coumaric acid, ferullic acid, butyl *p*-coumarate, pentyl p-coumarate, pentenyl *p*-coumarate, pinocembrin, pinobanksin, chrysin, galangin, and kaempferol	[54]
Greece	hydroethanolic	*Apis mellifera*	Pinocembbrin, apigenin, chrysin, galangin, ellagic acid, tectochrysin, syringic acid, ferullic acid, gallic acid, hesperetin, luteolin, *p*-coumaric acid, pinobanksin, caffeic acid, pinostrobin, caffeic acid phenethyl ester, quercetin, rhamnetin, kaempferol, chlorogenic acid, protocatechuic acid, kaempferide, acacetin, resveratrol, eriodictyol, naringenin, pinobanksin-3-O-acetate, catechin, and rutin	[37]
China	hydroethanolic	*Apis mellifera*	Caffeic acid, *p*-coumaric acid, ferulic acid, isoferulic acid, 3,4-dimethylcaffeic acid, pinobanksin, chrysin, pinocembrin, galangin, pinobanksin-3-acetate, and caffeic acid phenethyl ester	[38]
Taiwan	hydroethanolic	*Apis mellifera*	Propolin A-F (prenylflavanones)	[41]
India	hydroethanolic	*Apis mellifera*	Gallic acid, naringin, caffeic acid, *p*-coumaric acid, ferulic acid, quercetin, cinnamic acid, kaempferol, chrysin, galanginin, and caffeic acid phenethyl ester	[43]
Brazil	hydroethanolic	*Apis mellifera*	Artepillin C, kaempferol, kaempferide, *p*-coumaric acid, 3-prenyl-4-hydroxycinnamic acid, retusapurpurin A and B, formononetin, biochanin A, vestitol, neovestitol, daidzein, galangin, pinocembrin, chrysin, apigenin, and pinobanksin	[19,44,45,46,47,48,49,50,51,52]
Indonesia	hydroethanolic and chloroform	*Apis mellifera*	Alk(en)ylresorcinols, propolin C, D, F, and G	[42]
**Stingless bees**				
Malaysia	hydroethanolic	*Geniotrigona thoracia*	Caffeic acid, *p*-coumaric acid, quercetin, myricetin, naringenin, hesperitin, kaempferol, and baicaline	[55]
Malaysia	ethanolic	*Heterotrigona itama*	Gallic acid and its derivatives, caffeic acid and its derivatives, vanillic acid, syringic acid, protocatechuic acid, benzoic acid, vitexin-O-gallate, pinobanksin, lapachol, acetyleugenol, kaempferol, and mangostin	[56]
Thailand	hydroethanolic	*Tetrigona apicalis*	Gallic acid, eriodictyol, isoquercetin, quercetin, hydroquinin, catechin	[57]
India	ethanolic	*Not determined*	Gallic acid, naringin, caffeic acid, *p*-coumaric acid, ferullic acid, quercetin, cinnamic acid, kaempferol, and caffeic acid phenethyl ester	[58]
Brazil	hydroethanolic	*Frieseomelitta longipes*	Xanthochymol and gambogenone	[59]
Brazil	hydroethanolic	*Melipona subnitida*	Gallic acid and its derivatives, *p*-coumaric acid and its derivatives, cinnamic acid and its derivatives, kaempferol and its derivatives, quercetin and its derivatives, naringenin and its derivatives, ellagic acid, aromadendrin, myricetin dimethyl-ether, and herbacetin	[60]
Brazil	hydroethanolic hydroethanolic aqueous aqueous	*Melipona quadrifasciata* *Tetragonisca angustula* *Melipona quadrifasciata* *Tetragonisca angustula*	Quercetin, epigallocatechin, *p*-OH-benzoic acid, epigallocatecchin gallate, and coumaric acid Quercetin, *p*-OH-benzoic acid, caffeic acid, and coumaric acid Rutin, gallic acid, gallocatechin, epicatechin gallate, and syringic acid Quercetin, gallic acid, and gallocatechin	[61]
Brazil	hydroethanolic	*Melipona orbignyi*	Gallic acid and its derivatives, coumaric acid and its derivatives, aromadendrin, naringenin	[62]
Brazil	hydroethanolic	*Tetragonisca fiebrigi*	Benzoic acid, caffeic acid and its derivatives, cinnamic acid and its derivatives, *p*-coumaric acid and its derivatives	[63]
Brazil	hydroethanolic	*Melipona fasciculata*	Gallic acid and its derivatives, ellagic acid, and valoneic acid dilactone	[64]
Brazil	hydroethanolic	*Scaptotrigona Bipunctata* *Melipona quadrifasciata anthidioides*	Vicenin-1, -2, and -3Mepuberin	[65]
Australia	methanol, followed by diethyl ether and ethyl-O-acetate	*Tetragonula carbonaria*	Cinnamic acid, *p*-coumaric acid, phenolic acid, and gallic acid	[66]
Tanzania	hydroethanolic	*Meliponula ferruginea*	*p*-hydroxybenzoic acid, vanillic acid, *p*-coumaric acid, caffeic acid, resorcinol, cardanol, and anacardic acid	[67]

**Table 2:** The profile of terpenoids of propolis from various sources. These are for illustrative purposes only and by no means exhaustive.

Country	Extracts	Species	Terpenoid Profile	References
Italy	hydroethanolic	*Apis mellifera*	Diterpenic acids	[36]
Greece	hydroethanolic	*Apis mellifera*	α-pinene, junipene, and δ-cadinene, isocupressic acid, pimaric acid, communic acid, and 14,15-dinor-13-oxo-8(17)-labden-19-oic acid	[72,73]
Malta	hydroethanolic	*Apis mellifera*	2-acetoxy-6-p-methoxybenzoyljaeschkeanadiol, 2-acetoxy-6-p-methoxybenzoyljaeschkeanadiol, ferutinin, and teferin	[74]
Malaysia	hydroethanolic	*Geniotrigona thoracia*	fren-9(11)-en-2-alpha-ol, lup-20(29)-ene-3,21-dione, 28-hydroxy-, beta-amyrenol, and friedelan-y-al	[55]
Malaysia	hydroethanolic	*Tetrigona apicalis*	α-cubebene, copaene, caryophyllene, bicyclogermacrene, caryophyllene oxide, α-cadinol, α-amyrin, and β-amyrin	[81]
Malaysia	ethanolic	*Heterotrigona itama*	24(*E*)-cycloart-24-ene-26-ol-3-one, 20-hydroxy-24-dammaren-3-one,	[56]
Indonesia			mangiferolic acid, cycloartenol, and ambolic acid	[75]
Mexico	solid-phase microextraction	*Melipona beecheii*	(*Z*)-ocimenone, α-pinene, *m*-cymene, trans-isocarveol, limonene, verbenone, β-pinene, acampholenal, *m*-cymenene, trans-pinocamphone and trans-pulegol	[82]
Brazil	hydroethanolic	*Frieseomelitta longipes*	Pseudolimonene, β-phellandrene, (*Z*)-β-ocimene, α-cubebene, α-copaene, β-bourbonene, β-longipinene, α-gurjunene, α-cis-bergamotene, β-caryophyllene, β-copaene, β-trans-bergamotene, α-humulene, γ-muurolene, germacrene D, β-chamigrene, valencene, β-bisabolene, γ-cadinene, δ-cadinene, germacrene B	[59]
Brazil	hydroethanolic	*Melipona orbignyi*	Diterpenes, sequisterpenes, and triterpenes	[62]
Brazil	hydroethanolic	*Scaptotrigona bipunctata* *Melipona quadrifasciata anthidioides*	Triterpene (related to α-amyrin or β-amyrin) 7-O-methyl aromadendrin, abietic acid and its derivatives,	[65]
Australia	methanol, followed by diethyl ether and ethyl-O-acetate	*Tetragonula carbonaria*	Abietic acid, dehydroabietic acid, pimaric acid, and β-amyrin	[66]
Tanzania	hydroethanolic	*Meliponula ferruginea*	Diterpenic acid (pimaric), communic acid, 13-epi-cupressic acid, imbricataloic acid, abietic acid, dehydroabietic acid, acetylisocupressic acid, β-amyrin, cycloartenol, lupeol, β-amyrenone, triterpenic acid, triterpene acetate (betulin), lupenon, dammarenone, mangiferolic acid	[67]

**Table 3:** Antiviral properties of propolis.

Propolis/Propolis-Derived Compounds	Types of Virus	Mechanisms of Action	References
Chrysin, kaempferol, acacetin, galangin, and quercetin	herpesvirus, adenovirus, rotavirus, and coronavirus	Not determined	[89]
Kaempferol and *p*-coumaric acid	rhinovirus	Prevention of the entry of human rhinovirus and inhibition of the viral replication.	[112]
Liposomal propolis	Sars Cov 2	Interfering with 3CL-protease and S1 spike protein of Sars-Cov 2.	[15]
Withanone, caffeic acid phenethyl ester, sulabiroins A, (2S)-5,7-dihydroxy-4’-methoxy-8-prenylflavanone acid, glyasperin A, and broussoflavonol F	Sars Cov 2	Interfering with the highly conserved residues (substrate-binding pocket) of M^pro^ protein of Sars-Cov 2.	[93,94,113]
Isopentyl ferulate	influenza virus A (H3N2)	Not determined	[95]
Ethanolic extract of propolis	influenza virus A/PR/8/34 (H1N1)	Reducing the viral load in the bronchoalveolar lavage fluids of the lungs.	[96]
Apigenin, kaempferol, and coumaric acid	Influenza A/PR/8/34(H1N1) influenza A/Toyama/26/2011 (H1N1)	Reducing the viral load in the bronchoalveolar lavage fluids of the lungs.	[97]
Water extract of propolis, caffeoylquinic acids, and 3,4-dicaffeoylquinic acid	influenza virus A/WSN/33 (H1N1)	Increase in the expression of tumor necrosis factor-related apoptosis-inducing ligand (TRAIL) which expedited viral clearance.	[98,99]
Moronic acid	HIV	Inhibition of HIV in H9 lymphocytes.	[100]
Hydroalcoholic extract of propolis	HIV	Inhibition of HIV-1 infected CD4+ lymphocyte and microglial cell cultures.	[101]
Ethyl acetate extract of propolis, naringenin, quercetin, and diprenylcinnamic acid	HIV	Not determined	[102]
Galangin, kaempferol, and quercetin	Herpesvirus	Not determined	[119]
Aqueous and ethanolic extracts of propolis	Herpesvirus	Not determined	[104]
Poplar propolis extract (ACF^®^)	Herpesvirus	Not determined	[105]
Hydromethanolic extract of propolis, pyrrolizidine alkaloids, and C-glycosyl flavones	Herpesvirus	Not determined	[106]
Ethanolic extracts of propolis	Herpesvirus	Significant reduction of the viral load in brains and skins of mice infected with HSV-1.	[108]
Hydroalcoholic extract (70% ethanol) of propolis	Herpesvirus	Reduction of the severity of extravaginal lesions and histological damage in the vaginal tissue infected with HSV-2.	[109]
Ferulic acid	parvovirus	Inhibition and reversal of the parvovirus-induced expression of pro-apoptotic genes Bid, Bad, Bim, and Bak.	[114]
Quercetin and epigallocatechin gallate	Not determined	Zinc ionophore	[117]

**Table 4:** Antifungal properties of propolis.

Propolis/Propolis-Derived Compounds	Types of Fungi and Yeasts	Mechanisms of Action	References
Ethanolic extract of propolis	20 strains each of *Candida albicans*, *Candida tropicalis*, *Candida krusei* and 15 strainsof *Candida guilliermondii.*	Not determined	[141]
Ethanolic extract of propolis and propolis microparticles	clinical yeast isolates of vulvovaginal candidiasis	Not determined	[144]
n-hexane extract of propolis	*Candida* spp.	Not determined	[148]
Hydroethanolic extract of propolis	*C. parapsilosis*, *C. tropicalis*, *C. albicans*, and other species	Not determined	[149]
Methanolic extract of propolis, 2′,4′-dihydroxy-3-methoxychalcone and 2′,4′-dihydroxychalcone	*M. gypseum*, *T. mentagrophytes*, *and T. rubrum*	Not determined	[151]
Hydroethanolic extract of propolis	*C. albicans*, *T.rubrum*, and *A. fumigatus*	Not determined	[152]
Hydroethanolic, methanolic, aqueous, and dichloromethane extracts of propolis	*C. albicans* and *C. glabrata*	Not determined	[154]
Propolis Standardized Extract (EPP-AF^®^)	*C. abicans*	Induction of apoptosis through metacaspase and Ras signaling. Disruption the expression of various genes involved in pathogenesis, cell adhesion, biofilm formation, filamentous growth, and phenotypic switching.	[159]
Pinocembrin	*P. italicum*	Disruption of energy homeostasis, mycelia growth, the structure of the hyphae and the cell membrane. Reduction of the phosphorylated adenosine nucleotides levels.	[160]

**Table 5:** Antiparasitic properties of propolis.

Propolis/Propolis-Derived Compounds	Types of Parasites	Mechanisms of Action	References
Ethanolic extracts of propolis	*Trypanosomabrucei*, *Leishmania donovani*, *Plasmodium falciparum*, *Crithidiafasciculata* and *Mycobacterium marinum*	Not determined	[161]
Hydroethanolic, ethyl acetate, and dichloromethane extracts of propolis	Chloroquine (CQ)-sensitive *Plasmodium falciparum* 3D7 and *Plasmodium berghei* (ANKA strain)	Not determined	[162]
Methanolic extract of propolis	*P. chabaudin*	Increase in TNF-α and interferon-γ	[163]
Ethanolic extract of propolis and supercritically extracted propolis extract	*T. cruzi* Y strain	Not determined	[164]
Ethanolic extract of propolis liquiritigenin, pinocembrin, vestitol, medicarpin, 8-prenylnaringenin, 6-prenylnaringenin, propolin D, macarangin, and dihydrobenzofuran	Standarddrug-sensitive *T. brucei brucei* clone andtwo pentamidine-resistant types	Not determined	[166]
1,3,7-trihydroxy-2,8-di-(3-methylbut-2-enyl)xanthone, 1,3,7-trihydroxy-4,8-di-(3-methylbut-2-enyl)xanthone 1,7-dihydroxy-8-(3-methylbut-2-enyl)-3-(methylbut-2-enyloxy) xanthone, ambonic acid, mangiferonic acid and a mixture of α-amyrin with mangiferonic acid	*T. brucei brucei*	Not determined	[167]
β-phenethyl caffeate, farnesyl caffeate	*T. brucei brucei*	Not determined	[165]
Taxifolin-3-acetyl-4′-methyl ether and bilobol	*T. brucei*, *P. falciparum* *T. spiralis*,and *C. elegans*	Induction of cell lysis, disruption phospholipid metabolism and depletion of lipids such as phosphatidyl glycerol (PG) and phosphatidyl inositol (PI) lipids.	[179]
Rosmarinic acid and apigenin	*L. donovani*	Promotion of cell lysis, cytoplasmic condensation, and kinetoplast and nuclear DNA aggregation. Promotion of cell arrest at the G0/G1 phase and induced iron chelation.	[180]
Resveratrol	*T. vaginalis*	Disruption of hydrogenosome metabolism, by affecting [Fe]-hydrogenase (Tvhyd), pyruvate-ferredoxin oxidoreductase, and heat shock protein 70 (Hsp70).	[182]
Kaempferol	*Entamoeba histolityca*	Modification of the expression of actin, myosin II heavy chain and cortexillin II.	[185]
Epicathechin	*E. histolytica*	Disruption the expression of the heat shock protein 70, myosin II heavy chain, and actin. Disruption of the expression offructose-1,6-biphosphate aldolase and glyceraldehyde-phosphate dehydrogenase.	[186]
Apigenin and quercetin	*L. amazonensis*	Upregulation of the expression of reactive oxygen species (ROS), induction of mitochondrial dysfunction and membrane potential disruption, and the inhibition of ribonucleotide reductase.	[187,188,189]
Caffeic acid	*L. amazonensis*	Induction of morphological changes, disruption of the integrity of cellular plasma membrane and mitochondria, and consequently promotion ofapoptosis. Upregulation of the inflammatory response of macrophages by promoting the expression of ROS and TNF-α, while reducing the expression of IL-10 and the availability of iron.	[190]
Lupane, maslinic acid and ursolic acid, limonene, α-terpineol, 1,8-cineole, and linalool	*L. amazonensis*, *Toxoplasma gondii*, and *T. brucei*	Induction of morphological changes, promotion of apoptosis, and inhibition of crucial metabolic proteases and enzymes.	[191,193,194,195,196,200,201,202]

## Data Availability

Not applicable.

## References

[1] Tibenderana J.K., D’Alessandro U., Erhart A., Rosenthal P.J., Achan J., Yeka A., Baliraine F.N., Talisuna A.O. (2011). Quinine, an old anti-malarial drug in a modern world: Role in the treatment of malaria. Malar. J..

[2] (2015). Luz Yolanda Toro Suarez. Nobel Prize Physiol. Med..

[3] Fleming A. (1945). Penicillin. Nobel Lect..

[4] Niu G., Johnson R.M., Berenbaum M.R. (2011). Toxicity of mycotoxins to honeybees and its amelioration by propolis. Apidologie.

[5] Mao W., Schuler M.A., Berenbaum M.R. (2013). Honey constituents up-regulate detoxification and immunity genes in the western honey bee Apis mellifera. Proc. Natl. Acad. Sci. USA.

[6] Yemor T., Phiancharoen M., Eric Benbow M., Suwannapong G. (2015). Effects of stingless bee propolis on Nosema ceranae infected Asian honey bees, Apis cerana. J. Apic. Res..

[7] Borba R.S., Klyczek K.K., Mogen K.L., Spivak M. (2015). Seasonal benefits of a natural propolis envelope to honey bee immunity and colony health. J. Exp. Biol..

[8] Saelao P., Borba R.S., Ricigliano V., Spivak M., Simone-Finstrom M. (2020). Honeybee microbiome is stabilized in the presence of propolis. Biol. Lett..

[9] Dalenberg H., Maes P., Mott B., Anderson K.E., Spivak M. (2020). Propolis envelope promotes beneficial bacteria in the honey bee (Apis mellifera) mouthpart microbiome. Insects.

[10] Wang K., Ping S., Huang S., Hu L., Xuan H., Zhang C., Hu F. (2013). Molecular mechanisms underlying the in vitro anti-inflammatory effects of a flavonoid-rich ethanol extract from chinese propolis (poplar type). Evid. Based Complement. Altern. Med..

[11] Ibrahim N., Zakaria A.J., Ismail Z., Mohd K.S. (2016). Antibacterial and phenolic content of propolis produced by two Malaysian stingless bees, Heterotrigona itama and Geniotrigona thoracica. Int. J. Pharmacogn. Phytochem. Res..

[12] Corrêa F.R.S., Schanuel F.S., Moura-Nunes N., Monte-Alto-Costa A., Daleprane J.B. (2017). Brazilian red propolis improves cutaneous wound healing suppressing inflammation-associated transcription factor NFκB. Biomed. Pharmacother..

[13] Zhang W., Cai Y., Chen X., Ji T., Sun L. (2020). Optimized extraction based on the terpenoids of Heterotrigona itama propolis and their antioxidative and anti-inflammatory activities. J. Food Biochem..

[14] Amalia E., Diantini A., Subarnas A. (2020). Water-soluble propolis and bee pollen of Trigona spp. From South Sulawesi Indonesia induce apoptosis in the human breast cancer MCF-7 cell line. Oncol. Lett..

[15] Refaat H., Mady F.M., Sarhan H.A., Rateb H.S., Alaaeldin E. (2021). Optimization and evaluation of propolis liposomes as a promising therapeutic approach for COVID-19. Int. J. Pharm..

[16] Zulhendri F., Felitti R., Fearnley J., Ravalia M. (2021). The use of propolis in dentistry, oral health, and medicine: A review. J. Oral Biosci..

[17] Kuropatnicki A.K., Szliszka E., Krol W. (2013). Historical aspects of propolis research in modern times. Evid. Based Complement. Altern. Med..

[18] Ghisalberti E. (1979). Propolis: A review. Bee World.

[19] Park Y.K., Alencar S.M., Aguiar C.L. (2002). Botanical origin and chemical composition of Brazilian propolis. J. Agric. Food Chem..

[20] Sahinler N., Kaftanoglu O. (2005). Natural product propolis: Chemical composition. Nat. Prod. Res..

[21] Huang S., Zhang C.P., Wang K., Li G.Q., Hu F.L. (2014). Recent advances in the chemical composition of propolis. Molecules.

[22] Salatino A., Salatino M.L.F. (2021). Scientific note: Often quoted, but not factual data about propolis composition. Apidologie.

[23] Watanabe M.A.E., Amarante M.K., Conti B., Sforcin J.M. (2011). Cytotoxic constituents of propolis inducing anticancer effects: A review. J. Pharm. Pharmacol..

[24] Braakhuis A. (2019). Evidence on the health benefits of supplemental propolis. Nutrients.

[25] Zulhendri F., Ravalia M., Kripal K., Chandrasekaran K., Fearnley J., Perera C.O. (2021). Propolis in metabolic syndrome and its associated chronic diseases: A narrative review. Antioxidants.

[26] Lattanzio V., Kroon P.A., Quideau S., Treutter D. (2009). Plant phenolics—Secondary metabolites with diverse functions. Recent Adv. Polyphen. Res..

[27] Cheynier V., Comte G., Davies K.M., Lattanzio V., Martens S. (2013). Plant phenolics: Recent advances on their biosynthesis, genetics, andecophysiology. Plant Physiol. Biochem..

[28] Bhattacharya A., Sood P., Citovsky V. (2010). The roles of plant phenolics in defence and communication during Agrobacterium and Rhizobium infection. Mol. Plant Pathol..

[29] Quideau S., Deffieux D., Douat-Casassus C., Pouységu L. (2011). Plant polyphenols: Chemical properties, biological activities, and synthesis. Angew. Chemie Int. Ed..

[30] Lattanzio V., Ramawat K.G., Merillon J.M. (2013). Phenolic Compounds: Introduction. Natural Products.

[31] Singla R.K., Dubey A.K., Garg A., Sharma R.K., Fiorino M., Ameen S.M., Haddad M.A., Al-Hiary M. (2019). Natural polyphenols: Chemical classification, definition of classes, subcategories, and structures. J. AOAC Int..

[32] Kougan G.B., Tabopda T., Kuete V., Verpoorte R., Kuete V. (2013). Simple phenols, phenolic acids, and related esters from the medicinal plants of Africa. Medicinal Plant Research in Africa Pharmacology and Chemistry.

[33] Heleno S.A., Martins A., Queiroz M.J.R.P., Ferreira I.C.F.R. (2015). Bioactivity of phenolic acids: Metabolites versus parent compounds: A review. Food Chem..

[34] Kiokias S., Proestos C., Oreopoulou V. (2020). Phenolic acids of plant origin-a review on their antioxidant activity in vitro (O/W emulsion systems) along with their in vivo health biochemical properties. Foods.

[35] Umezawa T. (2003). Diversity in lignan biosynthesis. Phytochem. Rev..

[36] Bankova V., Popova M., Bogdanov S., Sabatini A.-G. (2002). Chemical composition of European propolis: Expected and unexpected results. Z. Naturforsch. C..

[37] Kasiotis K.M., Anastasiadou P., Papadopoulos A., Machera K. (2017). Revisiting Greek propolis: Chromatographic analysis and antioxidant activity study. PLoS ONE.

[38] Shi H., Yang H., Zhang X., Yu L. (2012). Identification and quantification of phytochemical composition and anti-inflammatory and radical scavenging properties of methanolic extracts of Chinese propolis. J. Agric. Food Chem..

[39] Chen C., Wu C., Shy H., Lin J. (2003). Cytotoxic prenylflavanones from Taiwanese propolis. J. Nat. Prod..

[40] Chen C.-N., Weng M.-S., Wu C.-L., Lin J.-K. (2004). Comparison of radical scavenging activity, cytotoxic effects and apoptosis induction in human melanoma cells by taiwanese propolis from different sources. Evid. Based Complement. Altern. Med..

[41] Chen Y.W., Wu S.W., Ho K.K., Lin S.B., Huang C.Y., Chen C.N. (2008). Characterisation of Taiwanese propolis collected from different locations and seasons. J. Sci. Food Agric..

[42] Trusheva B., Popova M., Koendhori E.B., Tsvetkova I., Naydenski C., Bankova V. (2011). Indonesian propolis: Chemical composition, biological activity and botanical origin. Nat. Prod. Res..

[43] Kasote D.M., Pawar M.V., Bhatia R.S., Nandre V.S., Gundu S.S., Jagtap S.D., Kulkarni M.V. (2017). HPLC, NMR based chemical profiling and biological characterisation of Indian propolis. Fitoterapia.

[44] De Oliveira P.F., De Souza Lima I.M., Munari C.C., Bastos J.K., Da Silva Filho A.A., Tavares D.C. (2014). Comparative evaluation of antiproliferative effects of brazilian green propolis, its main source baccharis dracunculifolia, and their major constituents artepillin C and baccharin. Planta Med..

[45] Szliszka E., Kucharska A.Z., Sokół-ŁȨtowska A., Mertas A., Czuba Z.P., Król W. (2013). Chemical composition and anti-inflammatory effect of ethanolic extract of Brazilian green propolis on activated J774A.1 macrophages. Evid. Based Complement. Altern. Med..

[46] De Carvalho C., Fernandes W.H.C., Mouttinho T.B.F., De Souza D.M., Marcucci M.C., D’Alpino P.H.P. (2019). Evidence-Based studies and perspectives of the use of brazilian green and red propolis in dentistry. Eur. J. Dent..

[47] Freires I.A., De Alencar S.M., Rosalen P.L. (2016). A pharmacological perspective on the use of Brazilian Red Propolis and its isolated compounds against human diseases. Eur. J. Med. Chem..

[48] Rufatto L.C., dos Santos D.A., Marinho F., Henriques J.A.P., Roesch Ely M., Moura S. (2017). Red propolis: Chemical composition and pharmacological activity. Asian Pac. J. Trop. Biomed..

[49] Regueira M.S., Tintino S.R., da Silva A.R.P., do Socorro Costa M., Boligon A.A., Matias E.F.F., de Queiroz Balbino V., Menezes I.R.A., Melo Coutinho H.D. (2017). Seasonal variation of Brazilian red propolis: Antibacterial activity, synergistic effect and phytochemical screening. Food Chem. Toxicol..

[50] de Freitas M.C.D., de Miranda M.B., de Oliveira D.T., Vieira-Filho S.A., Caligiorne R.B., de Figueiredo S.M. (2018). Biological activities of red propolis: A review. Recent Pat. Endocr. Metab. Immune Drug Discov..

[51] Curti V., Zaccaria V., Sokeng A.J.T., Dacrema M., Masiello I., Mascaro A., D’antona G., Daglia M. (2019). Bioavailability and in vivo antioxidant activity of a standardized polyphenol mixture extracted from brown propolis. Int. J. Mol. Sci..

[52] do Nascimento Araújo C., Mayworm M.A.S., Yatsuda R., Negri G., Salatino M.L.F., Salatino A., Timenetsky J., Campos G.B. (2020). Chemical composition and antimycoplasma activity of a brown propolis from southern Brazil. J. Food Sci. Technol..

[53] Popova M., Trusheva B., Bankova V. (2017). Content of biologically active compounds in Bulgarian propolis: A basis for its standardization. Bulg. Chem. Commun..

[54] Popova M., Giannopoulou E., Skalicka-Wózniak K., Graikou K., Widelski J., Bankova V., Kalofonos H., Sivolapenko G., Gaweł-Bȩben K., Antosiewicz B. (2017). Characterization and biological evaluation of propolis from Poland. Molecules.

[55] Nazir H., Shahidan W.N.S., Ibrahim H.A., Ismail T.N.N.T. (2018). Chemical constituents of Malaysian geniotrigona thoracica propolis. Pertanika J. Trop. Agric. Sci..

[56] Zhao L., Yu M., Sun M., Xue X., Wang T., Cao W., Sun L. (2017). Rapid determination of major compounds in the ethanol extract of geopropolis from Malaysian stingless bees, heterotrigona itama, by UHPLC-Q-TOF/MS and NMR. Molecules.

[57] Kraikongjit S., Jongjitvimol T., Mianjinda N., Sirithep N., Kaewbor T., Jumroon N., Jongjitvimol J. (2018). Antibacterial effect of plant resin collected fromTetrigona apicalis (Smith, 1857) in Thung Salaeng Luang National Park, Phitsanulok. Walailak J. Sci. Technol..

[58] Kasote D.M., Pawar M.V., Gundu S.S., Bhatia R., Nandre V.S., Jagtap S.D., Mahajan S.G., Kulkarni M.V. (2019). Chemical profiling, antioxidant, and antimicrobial activities of Indian stingless bees propolis samples. J. Apic. Res..

[59] De Souza E.C.A., Da Silva E.J.G., Cordeiro H.K.C., Lage Filho N.M., Da Silva F.M.A., Dos Reis D.L.S., Porto C., Pilau E.J., Da Costa L.A.M.A., De Souza A.D.L. (2018). Chemical compositions and antioxidant and antimicrobial activities of propolis produced by frieseomelitta longipes and apis mellifera BEES. Quim. Nova.

[60] de Sousa-Fontoura D.M.N., Olinda R.G., Viana G.A., Kizzy K.M., Batista J.S., Serrano R.M.O.T., Silva O.M.D., Camara C.A., Silva T.M.S. (2020). Wound healing activity and chemical composition of geopropolis from Melipona subnitida. Rev. Bras. Farmacogn..

[61] dos Santos L., Hochheim S., Boeder A.M., Kroger A., Tomazzoli M.M., Dal Pai Neto R., Maraschin M., Guedes A., de Cordova C.M.M. (2017). Chemical characterization, antioxidant, cytotoxic and antibacterial activity of propolis extracts and isolated compounds from the Brazilian stingless bees Melipona quadrifasciata and Tetragonisca angustula. J. Apic. Res..

[62] dos Santos H.F., Campos J.F., dos Santos C.M., Balestieri J.B.P., Silva D.B., Carollo C.A., de Picoli Souza K., Estevinho L.M., dos Santos E.L. (2017). Chemical profile and antioxidant, anti-inflammatory, antimutagenic and antimicrobial activities of geopropolis from the stingless bee Melipona orbignyi. Int. J. Mol. Sci..

[63] Campos J.F., Das Santos U.P., Da Rocha P.D.S., Damião M.J., Balestieri J.B.P., Cardoso C.A.L., Paredes-Gamero E.J., Estevinho L.M., De Picoli Souza K., Dos Santos E.L. (2015). Antimicrobial, antioxidant, anti-inflammatory, and cytotoxic activities of propolis from the stingless bee tetragonisca fiebrigi (Jataí). Evid. Based Complement. Altern. Med..

[64] Dutra R.P., De Barros Abreu B.V., Cunha M.S., Batista M.C.A., Torres L.M.B., Nascimento F.R.F., Ribeiro M.N.S., Guerra R.N.M. (2014). Phenolic acids, hydrolyzable tannins, and antioxidant activity of geopropolis from the stingless bee melipona fasciculata smith. J. Agric. Food Chem..

[65] Cisilotto J., Sandjo L.P., Faqueti L.G., Fernandes H., Joppi D., Biavatti M.W., Creczynski-Pasa T.B. (2018). Cytotoxicity mechanisms in melanoma cells and UPLC-QTOF/MS2 chemical characterization of two Brazilian stingless bee propolis: Uncommon presence of piperidinic alkaloids. J. Pharm. Biomed. Anal..

[66] Massaro F.C., Brooks P.R., Wallace H.M., Russell F.D. (2011). Cerumen of Australian stingless bees (Tetragonula carbonaria): Gas chromatography-mass spectrometry fingerprints and potential anti-inflammatory properties. Naturwissenschaften.

[67] Popova M., Gerginova D., Trusheva B., Simova S., Tamfu A.N., Ceylan O., Clark K., Bankova V. (2021). A preliminary study of chemical profiles of honey, cerumen, and propolis of the african stingless bee meliponula ferruginea. Foods.

[68] Gajger I.T., Dar S.A. (2021). Plant allelochemicals as sources of insecticides. Insects.

[69] Cheng A.X., Lou Y.G., Mao Y.B., Lu S., Wang L.J., Chen X.Y. (2007). Plant terpenoids: Biosynthesis and ecological functions. J. Integr. Plant Biol..

[70] Pichersky E., Raguso R.A. (2018). Why do plants produce so many terpenoid compounds?. New Phytol..

[71] Karunanithi P.S., Zerbe P. (2019). Terpene synthases as metabolic gatekeepers in the evolution of plant terpenoid chemical diversity. Front. Plant Sci..

[72] Melliou E., Stratis E., Chinou I. (2007). Volatile constituents of propolis from various regions of Greece—Antimicrobial activity. Food Chem..

[73] Popova M.P., Graikou K., Chinou I., Bankova V.S. (2010). GC-MS profiling of diterpene compounds in mediterranean propolis from Greece. J. Agric. Food Chem..

[74] Popova M., Trusheva B., Antonova D., Cutajar S., Mifsud D., Farrugia C., Tsvetkova I., Najdenski H., Bankova V. (2011). The specific chemical profile of Mediterranean propolis from Malta. Food Chem..

[75] Pujirahayu N., Suzuki T., Katayama T. (2019). Cycloartane-type triterpenes and botanical origin of propolis of stingless Indonesian bee tetragonula sapiens. Plants.

[76] Iqbal M., Fan T.P., Watson D., Alenezi S., Saleh K., Sahlan M. (2019). Preliminary studies: The potential anti-angiogenic activities of two Sulawesi Island (Indonesia) propolis and their chemical characterization. Heliyon.

[77] Wen C.C., Kuo Y.H., Jan J.T., Liang P.H., Wang S.Y., Liu H.G., Lee C.K., Chang S.T., Kuo C.J., Lee S.S. (2007). Specific plant terpenoids and lignoids possess potent antiviral activities against severe acute respiratory syndrome coronavirus. J. Med. Chem..

[78] Guimarães A.C., Meireles L.M., Lemos M.F., Guimarães M.C.C., Endringer D.C., Fronza M., Scherer R. (2019). Antibacterial activity of terpenes and terpenoids present in essential oils. Molecules.

[79] Wang C.Y., Chen Y.W., Hou C.Y. (2019). Antioxidant and antibacterial activity of seven predominant terpenoids. Int. J. Food Prop..

[80] Chen Y., Zhu Z., Chen J., Zheng Y., Limsila B., Lu M., Gao T., Yang Q., Fu C., Liao W. (2021). Terpenoids from Curcumae Rhizoma: Their anticancer effects and clinical uses on combination and versus drug therapies. Biomed. Pharmacother..

[81] Mohamed W.A.S., Ismail N.Z., Omar E.A., Abdul Samad N., Adam S.K., Mohamad S. (2020). GC-MS evaluation, antioxidant content, and cytotoxic activity of propolis extract from peninsular malaysian stingless bees, tetrigona apicalis. Evid. Based Complement. Altern. Med..

[82] Torres-González A., López-Rivera P., Duarte-Lisci G., López-Ramírez Á., Correa-Benítez A., Rivero-Cruz J.F. (2016). Analysis of volatile components from Melipona beecheii geopropolis from Southeast Mexico by headspace solid-phase microextraction. Nat. Prod. Res..

[83] Eroglu N., Akkus S., Yaman M., Asci B., Silici S. (2016). Amino acid and vitamin content of propolis collected by native caucasican honeybees. J. Apic. Sci..

[84] Tosic S., Stojanovic G., Mitic S., Pavlovic A., Alagic S. (2017). Mineral composition of selected serbian propolis samples. J. Apic. Sci..

[85] Wezgowiec J., Wieczynska A., Wieckiewicz W., Kulbacka J., Saczko J., Pachura N., Wieckiewicz M., Gancarz R., Wilk K.A. (2020). Polish propolis-Chemical composition and biological effects in tongue cancer cells and macrophages. Molecules.

[86] Dezmirean D.S., Paşca C., Moise A.R., Bobiş O. (2021). Plant sources responsible for the chemical composition and main bioactive properties of poplar-type propolis. Plants.

[87] Wolska K., Górska A., Antosik K., Ługowska K. (2019). Immunomodulatory effects of propolis and its components on basic immune cell functions. Indian J. Pharm. Sci..

[88] Al-Hariri M. (2019). Immune’s-boosting agent: Immunomodulation potentials of propolis. J. Fam. Community Med..

[89] Debiaggi M., Tateo F., Pagani L., Luini M., Romero E. (1990). Effects of propolis flavonoids on virus infectivity and replication. Microbiologica.

[90] Forouzanfar M.H., Afshin A., Alexander L.T., Biryukov S., Brauer M., Cercy K., Charlson F.J., Cohen A.J., Dandona L., Estep K. (2016). Global, regional, and national comparative risk assessment of 79 behavioural, environmental and occupational, and metabolic risks or clusters of risks, 1990–2015: A systematic analysis for the Global Burden of Disease Study 2015. Lancet.

[91] Harisna A.H., Nurdiansyah R., Syaifie P.H., Nugroho D.W., Saputro K.E., Firdayani, Prakoso C.D., Rochman N.T., Maulana N.N., Noviyanto A. (2021). In silico investigation of potential inhibitors to main protease and spike protein of SARS-CoV-2 in propolis. Biochem. Biophys. Reports.

[92] Khayrani A.C., Irdiani R., Aditama R., Pratami D.K., Lischer K., Ansari M.J., Chinnathambi A., Alharbi S.A., Almoallim H.S., Sahlan M. (2021). Evaluating the potency of Sulawesi propolis compounds as ACE-2 inhibitors through molecular docking for COVID-19 drug discovery preliminary study. J. King Saud Univ. Sci..

[93] Dewi L.K., Sahlan M., Pratami D.K., Agus A., Agussalim, Sabir A. (2021). Identifying propolis compounds potential to be covid-19 therapies by targeting sars-cov-2 main protease. Int. J. Appl. Pharm..

[94] Sahlan M., Irdiani R., Flamandita D., Aditama R., Alfarraj S., Ansari M.J., Khayrani A.C., Pratami D.K., Lischer K. (2021). Molecular interaction analysis of Sulawesi propolis compounds with SARS-CoV-2 main protease as preliminary study for COVID-19 drug discovery. J. King Saud Univ. Sci..

[95] Serkedjieva J., Manolova N., Bankova V. (1992). Anti-influenza virus effect of some propolis constituents and their analogues (esters of substituted cinnamic acids). J. Nat. Prod..

[96] Shimizu T., Hino A., Tsutsumi A., Yong K.P., Watanabe W., Kurokawa M. (2008). Anti-influenza virus activity of propolis in vitro and its efficacy against influenza infection in mice. Antivir. Chem. Chemother..

[97] Kai H., Obuchi M., Yoshida H., Watanabe W., Tsutsumi S., Park Y.K., Matsuno K., Yasukawa K., Kurokawa M. (2014). In vitro and in vivo anti-influenza virus activities of flavonoids and related compounds as components of Brazilian propolis (AF-08). J. Funct. Foods.

[98] Kuwata K., Urushisaki T., Takemura T., Tazawa S., Fukuoka M., Hosokawa-Muto J., Araki Y. (2011). Caffeoylquinic acids are major constituents with potent anti-influenza effects in brazilian green propolis water extract. Evid. Based Complement. Altern. Med..

[99] Kuwata K., Takemura T., Urushisaki T., Fukuoka M., Hosokawa-Muto J., Hata T., Okuda Y., Hori S., Tazawa S., Araki Y. (2012). 3,4-dicaffeoylquinic acid, a major constituent of Brazilian propolis, increases TRAIL expression and extends the lifetimes of mice infected with the influenza a virus. Evid. Based Complement. Altern. Med..

[100] Ito J., Chang F.R., Wang H.K., Park Y.K., Ikegaki M., Kilgore N., Lee K.H. (2001). Anti-AIDS agents. 48. Anti-HIV activity of moronic acid derivatives and the new melliferone-related triterpenoid isolated from Brazilian propolis. J. Nat. Prod..

[101] Gekker G., Hu S., Spivak M., Lokensgard J.R., Peterson P.K. (2005). Anti-HIV-1 activity of propolis in CD4+ lymphocyte and microglial cell cultures. J. Ethnopharmacol..

[102] da Silva C.C.F., Salatino A., da Motta L.B., Negri G., Salatino M.L.F. (2019). Chemical characterization, antioxidant and anti-HIV activities of a Brazilian propolis from Ceará state. Rev. Bras. Farmacogn..

[103] Amoros M., Sauvager F., Girre L., Cormier M. (1992). In vitro antiviral activity of propolis. Apidologie.

[104] Schnitzler P., Neuner A., Nolkemper S., Zundel C., Nowack H., Sensch K.H., Reichling J. (2010). Antiviral activity and mode of action of propolis extracts and selected compounds. Phyther. Res..

[105] Bankova V., Galabov A.S., Antonova D., Vilhelmova N., Di Perri B. (2014). Chemical composition of Propolis Extract ACF^®^ and activity against herpes simplex virus. Phytomedicine.

[106] Coelho G.R., Mendonça R.Z., De SennaVilar K., Figueiredo C.A., Badari J.C., Taniwaki N., Namiyama G., De Oliveira M.I., Curti S.P., Evelyn Silva P. (2015). Antiviral action of hydromethanolic extract of geopropolis from scaptotrigona postica against antiherpes simplex virus (HSV-1). Evid. Based Complement. Altern. Med..

[107] Hochheim S., Guedes A., Faccin-Galhardi L., Rechenchoski D.Z., Nozawa C., Linhares R.E., da Filho H.H.S., Rau M., Siebert D.A., Micke G. (2019). Determination of phenolic profile by HPLC–ESI-MS/MS, antioxidant activity, in vitro cytotoxicity and anti-herpetic activity of propolis from the Brazilian native bee Melipona quadrifasciata. Rev. Bras. Farmacogn..

[108] Kurokawa M., Shimizu T., Takeshita Y., Takamori Y., Kai H., Sawamura R., Yoshida H., Watanabe W., Tsutsumi A., Park Y.K. (2011). Efficacy of Brazilian propolis against herpes simplex virus type 1 infection in mice and their modes of antiherpetic efficacies. Evid. Based Complement. Altern. Med..

[109] Sartori G., Pesarico A.P., Pinton S., Dobrachinski F., Roman S.S., Pauletto F., Rodrigues L.C., Prigol M. (2012). Protective effect of brown Brazilian propolis against acute vaginal lesions caused by herpes simplex virus type 2 in mice: Involvement of antioxidant and anti-inflammatory mechanisms. Cell Biochem. Funct..

[110] Búfalo M.C., Figueiredo A.S., De Sousa J.P.B., Candeias J.M.G., Bastos J.K., Sforcin J.M. (2009). Anti-poliovirus activity of Baccharis dracunculifolia and propolis by cell viability determination and real-time PCR. J. Appl. Microbiol..

[111] Coelho G.R., De Senna Villar K., Figueiredo C.A., Badari J.C., Mendonca R.M.Z., Oliveira M.I., Curti S.P., Silva P.E.S., Do Nascimento R.M., Mendonca R.Z. (2014). Antiviral effects of Scaptotrigona postica propolis and their fractions. BMC Proc..

[112] Kwon M.J., Shin H.M., Perumalsamy H., Wang X., Ahn Y.J. (2020). Antiviral effects and possible mechanisms of action of constituents from Brazilian propolis and related compounds. J. Apic. Res..

[113] Kumar V., Dhanjal J.K., Kaul S.C., Wadhwa R., Sundar D. (2020). Withanone and caffeic acid phenethyl ester are predicted to interact with main protease (Mpro) of SARS-CoV-2 and inhibit its activity. J. Biomol. Struct. Dyn..

[114] Ma X., Guo Z., Zhang Z., Li X., Wang X., Liu Y., Wang X. (2020). Ferulic acid isolated from propolis inhibits porcine parvovirus replication potentially through Bid-mediate apoptosis. Int. Immunopharmacol..

[115] Hayakari R., Matsumiya T., Xing F., Tayone J.C., Dempoya J., Tatsuta T., Aizawa-Yashiro T., Imaizumi T., Yoshida H., Satoh K. (2013). Effects of Brazilian green propolis on double-stranded RNA-mediated induction of interferon-inducible gene and inhibition of recruitment of polymorphonuclear cells. J. Sci. Food Agric..

[116] Verhelst J., Hulpiau P., Saelens X. (2013). Mx proteins: Antiviral gatekeepers that restrain the uninvited. Microbiol. Mol. Biol. Rev..

[117] Dabbagh-Bazarbachi H., Clergeaud G., Quesada I.M., Ortiz M., O’Sullivan C.K., Fernández-Larrea J.B. (2014). Zinc ionophore activity of quercetin and epigallocatechin-gallate: From hepa 1-6 cells to a liposome model. J. Agric. Food Chem..

[118] Kaushik N., Subramani C., Anang S., Muthumohan R., Shalimar, Nayak B., Ranjith-Kumar C.T., Surjit M. (2017). Zinc salts block hepatitis E virus replication by inhibiting the activity of viral RNA-dependent RNA polymerase. J. Virol..

[119] Amoros M., Simōes C.M.O., Girre L., Sauvager F., Cormier M. (1992). Synergistic effect of flavones and flavonols against herpes simplex virus type 1 in cell culture. Comparison with the antiviral activity of propolis. J. Nat. Prod..

[120] Przybyłek I., Karpiński T.M. (2019). Antibacterial properties of propolis. Molecules.

[121] Almuhayawi M.S. (2020). Propolis as a novel antibacterial agent. Saudi J. Biol. Sci..

[122] Silva-Carvalho R., Baltazar F., Almeida-Aguiar C. (2015). Propolis: A complex natural product with a plethora of biological activities that can be explored for drug development. Evid. Based Complement. Altern. Med..

[123] Sforcin J.M., Bankova V. (2011). Propolis: Is there a potential for the development of new drugs?. J. Ethnopharmacol..

[124] Bogdan Kędzia E.H.-K. (2013). Aktywność antybiotyczna propolisu krajowego i europejskiego. The antibiotic activity of native and european propolis. Postępy Fitoter..

[125] Bridi R., Montenegro G., Nuñez-Quijada G., Giordano A., Fernanda Morán-Romero M., Jara-Pezoa I., Speisky H., Atala E., López-Alarcón C. (2015). International regulations of propolis quality: Required assays do not necessarily reflect their polyphenolic-related in vitro activities. J. Food Sci..

[126] Pamplona-Zomenhan L.C., Pamplona B.C., da Silva C.B., Marcucci M.C., Mimica L.M.J. (2011). Evaluation of the in vitro antimicrobial activity of an ethanol extract of Brazilian classified propolis on strains of Staphylococcus aureus. Brazilian J. Microbiol..

[127] Ivana Tlak G., Iva P., Mirza B., Ivan K., Siniša S., Toni V., Josipa V. (2017). Components responsible for antimicrobial activity of propolis from continental and Mediterranean regions in Croatian. Czech J. Food Sci..

[128] Šuran J., Cepanec I., Mašek T., Radić B., Radić S., Tlak Gajger I., Vlainić J. (2021). Propolis extract and its bioactive compounds—From traditional to modern extraction technologies. Molecules.

[129] Oksuz H., Duran N., Tamer C., Cetin M., Silici S. (2005). Effect of propolis in the treatment of experimental Staphylococcus aureus Keratitis in Rabbits. Ophthalmic Res..

[130] Orsi R.O., Fernandes A., Bankova V., Sforcin J.M. (2012). The effects of Brazilian and Bulgarian propolis in vitro against Salmonella Typhi and their synergism with antibiotics acting on the ribosome. Nat. Prod. Res..

[131] Al-Waili N., Al-Ghamdi A., Ansari M.J., Al-Attal Y., Salom K. (2012). Synergistic effects of honey and propolis toward drug multi-resistant Staphylococcus Aureus, Escherichia coli and Candida Albicans isolates in single and polymicrobial cultures. Int. J. Med. Sci..

[132] Kowacz M., Pollack G.H. (2020). Propolis-induced exclusion of colloids: Possible new mechanism of biological action. Colloids Interface Sci. Commun..

[133] Kowacz M., Pollack G.H. (2020). Cells in new light: Ion concentration, voltage, and pressure gradients across a hydrogel membrane. ACS Omega.

[134] Sharaf S., Higazy A., Hebeish A. (2013). Propolis induced antibacterial activity and other technical properties of cotton textiles. Int. J. Biol. Macromol..

[135] Abramiuc D., Ciobanu L., Muresan R., Chiosac M., Muresan A. (2013). Antibacterial finishing of cotton fabrics using biologically active natural compounds. Fibers Polym..

[136] Arıkan H.K., Solak H.H. (2017). Propolis Extract-PVA nanocomposites of textile design: Antimicrobial effect on gram positive and negative bacterias. Int. J. Second. Metab..

[137] Cheng Y., Moraru C.I. (2018). Long-range interactions keep bacterial cells from liquid-solid interfaces: Evidence of a bacteria exclusion zone near Nafion surfaces and possible implications for bacterial attachment. Colloids Surf. B Biointerfaces.

[138] Dias L.G., Pereira A.P., Estevinho L.M. (2012). Comparative study of different Portuguese samples of propolis: Pollinic, sensorial, physicochemical, microbiological characterization and antibacterial activity. Food Chem. Toxicol..

[139] Halder S., Yadav K.K., Sarkar R., Mukherjee S., Saha P., Haldar S., Karmakar S., Sen T. (2015). Alteration of Zeta potential and membrane permeability in bacteria: A study with cationic agents. Springerplus.

[140] Kujumgiev A., Tsvetkova I., Serkedjieva Y., Bankova V., Christov R., Popov S. (1999). Antibacterial, antifungal and antiviral activity of propolis of different geographic origin. J. Ethnopharmacol..

[141] Ota C., Unterkircher C., Fantinato V., Shimizu M.T. (2001). Antifungal activity of propolis on different species of Candida. Mycoses.

[142] Murad J.M., Calvi S.A., Soares A.M.V.C., Bankova V., Sforcin J.M. (2002). Effects of propolis from Brazil and Bulgaria on fungicidal activity of macrophages against Paracoccidioides brasiliensis. J. Ethnopharmacol..

[143] Siqueira A.B.S., Gomes B.S., Cambuim I., Maia R., Abreu S., Souza-Motta C.M., De Queiroz L.A., Porto A.L.F. (2009). Trichophyton species susceptibility to green and red propolis from Brazil. Lett. Appl. Microbiol..

[144] Bruschi M.L., Dota K.F.D., Consolaro M.E.L., Svidzinski T.I.E. (2011). Antifungal activity of brazilian propolis microparticles against yeasts isolated from vulvovaginal candidiasis. Evid. Based Complement. Altern. Med..

[145] Bonvehí J.S., Gutiérrez A.L. (2012). The antimicrobial effects of propolis collected in different regions in the Basque Country (Northern Spain). World J. Microbiol. Biotechnol..

[146] Mutlu Sariguzel F., Berk E., Koc A.N., Sav H., Demir G. (2016). Antifungal activity of propolis against yeasts isolated from blood culture: In vitro evaluation. J. Clin. Lab. Anal..

[147] Ghaly M.F., Ezzat S.M., Sarhan M.M. (1998). Use of propolis and ultragriseofulvin to inhibit aflatoxigenic fungi. Folia Microbiol..

[148] Pippi B., Lana A.J.D., Moraes R.C., Güez C.M., Machado M., de Oliveira L.F.S., Lino von Poser G., Fuentefria A.M. (2015). In vitro evaluation of the acquisition of resistance, antifungal activity and synergism of Brazilian red propolis with antifungal drugs on Candida spp.. J. Appl. Microbiol..

[149] Oliveira A.C.P., Shinobu C.S., Longhini R., Franco S.L., Svidzinski T.I.E. (2006). Antifungal activity of propolis extract against yeasts isolated from onychomycosis lesions. Mem. Inst. Oswaldo Cruz.

[150] Quiroga E.N., Sampietro D.A., Soberón J.R., Sgariglia M.A., Vattuone M.A. (2006). Propolis from the northwest of Argentina as a source of antifungal principles. J. Appl. Microbiol..

[151] Agüero M.B., Gonzalez M., Lima B., Svetaz L., Sánchez M., Zacchino S., Feresin G.E., Schmeda-Hirschmann G., Palermo J., Daniel Wunderlin A.N.D. (2010). Argentinean propolis from Zuccagnia punctata cav. (Caesalpinieae) exudates: Phytochemical characterization and antifungal activity. J. Agric. Food Chem..

[152] Falcão S.I., Vale N., Cos P., Gomes P., Freire C., Maes L., Vilas-Boas M. (2014). In vitro evaluation of portuguese propolis and floral sources for antiprotozoal, antibacterial and antifungal activity. Phyther. Res..

[153] Szweda P., Gucwa K., Kurzyk E., Romanowska E., Dzierżanowska-Fangrat K., Zielińska Jurek A., Kuś P.M., Milewski S. (2015). Essential oils, silver nanoparticles and propolis as alternative agents against fluconazole resistant candida albicans, candida glabrata and candida krusei clinical isolates. Indian J. Microbiol..

[154] Boisard S., Le Ray A.M., Landreau A., Kempf M., Cassisa V., Flurin C., Richomme P. (2015). Antifungal and antibacterial metabolites from a French poplar type propolis. Evid. Based Complement. Altern. Med..

[155] Berretta A.A., De Castro P.A., Cavalheiro A.H., Fortes V.S., Bom V.P., Nascimento A.P., Marquele-Oliveira F., Pedrazzi V., Ramalho L.N.Z., Goldman G.H. (2013). Evaluation of mucoadhesive gels with propolis (EPP-AF) in preclinical treatment of candidiasis vulvovaginal infection. Evid. Based Complement. Altern. Med..

[156] Bonfim A.P., Sakita K.M., Faria D.R., Arita G.S., Vendramini F.A.V.R., Capoci I.R.G., Braga A.G., dos Santos R.S., Bruschi M.L., Becker T.C.A. (2020). Preclinical approaches in vulvovaginal candidiasis treatment with mucoadhesive thermoresponsive systems containing propolis. PLoS ONE.

[157] Wagh V.D. (2013). Propolis: A wonder bees product and its pharmacological potentials. Adv. Pharmacol. Sci..

[158] Banskota A.H., Tezuka Y., Kadota S. (2001). Recent progress in pharmacological research of propolis. Phyther. Res..

[159] De Castro P.A., Bom V.L.P., Brown N.A., de Almeida R.S.C., Ramalho L.N.Z., Savoldi M., Goldman M.H.S., Berretta A.A., Goldman G.H. (2013). Identification of the cell targets important for propolis-induced cell death in Candida albicans. Fungal Genet. Biol..

[160] Peng L., Yang S., Cheng Y.J., Chen F., Pan S., Fan G. (2012). Antifungal activity and action mode of pinocembrin from propolis against Penicillium italicum. Food Sci. Biotechnol..

[161] Siheri W., Zhang T., Ebiloma G.U., Biddau M., Woods N., Hussain M.Y., Clements C.J., Fearnley J., Edrada Ebel R.A., Paget T. (2016). Chemical and antimicrobial profiling of propolis from different regions within Libya. PLoS ONE.

[162] Afrouzan H., Zakeri S., Mehrizi A.A., Molasalehi S., Tahghighi A., Shokrgozar M.A., Es-Haghi A., Djadid N.D. (2017). Anti-plasmodial assessment of four different Iranian propolis extracts. Arch. Iran. Med..

[163] AlGabbani Q., Mansour L., Elnakady Y.A., Al-Quraishy S., Alomar S., Al-Shaebi E.M., Abdel-Baki A.A.S. (2017). In vivo assessment of the antimalarial and spleen-protective activities of the Saudi propolis methanolic extract. Parasitol. Res..

[164] Silva R.P.D., Machado B.A.S., De Abreu Barreto G., Costa S.S., Andrade L.N., Amaral R.G., Carvalho A.A., Padilha F.F., Barbosa J.D.V., Umsza-Guez M.A. (2017). Antioxidant, antimicrobial, antiparasitic, and cytotoxic properties of various Brazilian propolis extracts. PLoS ONE.

[165] Otoguro K., Iwatsuki M., Ishiyama A., Namatame M., Nishihara-Tsukashima A., Kiyohara H., Hashimoto T., Asakawa Y., O’Mura S., Yamada H. (2012). In vitro antitrypanosomal activity of some phenolic compounds from propolis and lactones from Fijian Kawa (Piper methysticum). J. Nat. Med..

[166] Omar R.M.K., Igoli J., Gray A.I., Ebiloma G.U., Clements C., Fearnley J., Edrada Ebel R.A., Zhang T., De Koning H.P., Watson D.G. (2016). Chemical characterisation of Nigerian red propolis and its biological activity against Trypanosoma Brucei. Phytochem. Anal..

[167] Omar R., Igoli J.O., Zhang T., Gray A.I., Ebiloma G.U., Clements C.J., Fearnley J., Ebel R.A.E., Paget T., De Koning H.P. (2017). The chemical characterization of nigerian propolis samples and their activity against trypanosoma brucei. Sci. Rep..

[168] Gressler L.T., Da Silva A.S., Machado G., Rosa L.D., Dorneles F., Gressler L.T., Oliveira M.S., Zanette R.A., de Vargas A.C.P., Monteiro S.G. (2012). Susceptibility of Trypanosoma evansi to propolis extract in vitro and in experimentally infected rats. Res. Vet. Sci..

[169] Nweze N.E., Okoro H.O., Al Robaian M., Omar R.M.K., Tor-Anyiin T.A., Watson D.G., Igoli J.O. (2017). Effects of Nigerian red propolis in rats infected with Trypanosoma brucei brucei. Comp. Clin. Path..

[170] da Silveira Regueira-Neto M., Tintino S.R., Rolón M., Coronal C., Vega M.C., de Queiroz Balbino V., de Melo Coutinho H.D. (2018). Antitrypanosomal, antileishmanial and cytotoxic activities of Brazilian red propolis and plant resin of Dalbergia ecastaphyllum (L) Taub. Food Chem. Toxicol..

[171] Alotaibi A., Ebiloma G.U., Williams R., Alenezi S., Donachie A.M., Guillaume S., Igoli J.O., Fearnley J., de Koning H.P., Watson D.G. (2019). European propolis is highly active against trypanosomatids including Crithidia fasciculata. Sci. Rep..

[172] Alanazi S., Alenzi N., Alenazi F., Tabassum H., Watson D. (2021). Chemical characterization of Saudi propolis and its antiparasitic and anticancer properties. Sci. Rep..

[173] Pontin K., Da Silva Filho A.A., Santos F.F., Silva M.L.A.E., Cunha W.R., Nanayakkara N.P.D., Bastos J.K., De Albuquerque S. (2008). In vitro and in vivo antileishmanial activities of a Brazilian green propolis extract. Parasitol. Res..

[174] Hegazi A.G., El-fadaly H.A., Barakat A.M., Abou-el-doubal S.K.A. (2014). In vitro Effects of Some Bee Products on T. gondii Tachyzoites. Glob. Vet..

[175] Freitas S.F., Shinohara L., Sforcin J.M., Guimarães S. (2006). In vitro effects of propolis on Giardia duodenalis trophozoites. Phytomedicine.

[176] Mokhtar A.B., El-Gayar E.K., Habib E.S. (2016). In vitro anti-protozoal activity of propolis extract and cysteine proteases inhibitor (phenyl vinyl sulfone) on blastocystis species. J. Egypt. Soc. Parasitol..

[177] Asfaram S., Fakhar M., Keighobadi M., Akhtari J. (2020). Promising anti-protozoan activities of propolis (bee glue) as natural product: A review. Acta Parasitol..

[178] Fidalgo L.M., Ramos I.S., Parra M.G., Cuesta-Rubio O., Hernández I.M., Fernández M.C., Piccinelli A.L., Rastrelli L. (2011). Activity of Cuban propolis extracts on Leishmania amazonensis and Trichomonas vaginalis. Nat. Prod. Commun..

[179] Siheri W., Ebiloma G.U., Igoli J.O., Gray A.I., Biddau M., Akrachalanont P., Alenezi S., Alwashih M.A., Edrada-Ebel R.A., Muller S. (2019). Isolation of a novel flavanonol and an alkylresorcinol with highly potent anti-trypanosomal activity from libyan propolis. Molecules.

[180] Antwi C.A., Amisigo C.M., Adjimani J.P., Gwira T.M. (2019). In vitro activity and mode of action of phenolic compounds on leishmania donovani. PLoS Negl. Trop. Dis..

[181] Volpi N. (2004). Separation of flavonoids and phenolic acids from propolis by capillary zone electrophoresis. Electrophoresis.

[182] Mallo N., Lamas J., Leiro J.M. (2013). Hydrogenosome metabolism is the key target for antiparasitic activity of resveratrol against trichomonas vaginalis. Antimicrob. Agents Chemother..

[183] Duca A., Sturza A., Moacă E.A., Negrea M., Lalescu V.D., Lungeanu D., Dehelean C.A., Muntean D.M., Alexa E. (2019). Identification of resveratrol as bioactive compound of propolis from western Romania and characterization of phenolic profile and antioxidant activity of ethanolic extracts. Molecules.

[184] Embley T.M., Van Der Giezen M., Horner D.S., Dyal P.L., Foster P., Tielens A.G.M., Martin W., Tovar J., Douglas A.E., Cavalier-Smith T. (2003). Mitochondria and hydrogenosomes are two forms of the same fundamental organelle. Philos. Trans. R. Soc. B Biol. Sci..

[185] Bolaños V., Díaz-Martínez A., Soto J., Marchat L.A., Sanchez-Monroy V., Ramírez-Moreno E. (2015). Kaempferol inhibits Entamoeba histolytica growth by altering cytoskeletal functions. Mol. Biochem. Parasitol..

[186] Bolaños V., Díaz-Martínez A., Soto J., Rodríguez M.A., López-Camarillo C., Marchat L.A., Ramírez-Moreno E. (2014). The flavonoid (-)-epicatechin affects cytoskeleton proteins and functions in Entamoeba histolytica. J. Proteomics.

[187] Fonseca-Silva F., Canto-Cavalheiro M.M., Menna-Barreto R.F.S., Almeida-Amaral E.E. (2015). Effect of apigenin on leishmania amazonensis is associated with reactive oxygen species production followed by mitochondrial dysfunction. J. Nat. Prod..

[188] Fonseca-Silva F., Inacio J.D.F., Canto-Cavalheiro M.M., Almeida-Amaral E.E. (2011). Reactive oxygen species production and mitochondrial dysfunction contribute to quercetin induced death in Leishmania amazonensis. PLoS ONE.

[189] Sen G., Mukhopadhyay S., Ray M., Biswas T. (2008). Quercetin interferes with iron metabolism in Leishmania donovani and targets ribonucleotide reductase to exert leishmanicidal activity. J. Antimicrob. Chemother..

[190] da Silva Bortoleti B.T., Tomiotto-Pellissier F., Gonçalves M.D., Miranda-Sapla M.M., Assolini J.P., Carloto A.C., Lima D.M., Silveira G.F., Almeida R.S., Costa I.N. (2019). Caffeic acid has antipromastigote activity by apoptosis-like process; and anti-amastigote by TNF-α/ROS/NO production and decreased of iron availability. Phytomedicine.

[191] Teles C.B.G., Moreira-Dill L.S., de Almeida Silva A., Facundo V.A., de Azevedo W.F., da Silva L.H.P., Motta M.C.M., Stábeli R.G., Silva-Jardim I. (2015). A lupane-triterpene isolated from Combretum leprosum Mart. fruit extracts that interferes with the intracellular development of Leishmania (L.) amazonensis in vitro. BMC Complement. Altern. Med..

[192] Sanpa S., Popova M., Bankova V., Tunkasiri T., Eitssayeam S., Chantawannakul P. (2015). Antibacterial compounds from propolis of Tetragonula laeviceps and Tetrigona melanoleuca (Hymenoptera: Apidae) from Thailand. PLoS ONE.

[193] De Pablos L.M., González G., Rodrigues R., García Granados A., Parra A., Osuna A. (2010). Action of a pentacyclic triterpenoid, maslinic acid, against Toxoplasma gondii. J. Nat. Prod..

[194] Moneriz C., Mestres J., Bautista J.M., Diez A., Puyet A. (2011). Multi-targeted activity of maslinic acid as an antimalarial natural compound. FEBS J..

[195] Bero J., Beaufay C., Hannaert V., Hérent M.F., Michels P.A., Quetin-Leclercq J. (2013). Antitrypanosomal compounds from the essential oil and extracts of Keetia leucantha leaves with inhibitor activity on Trypanosoma brucei glyceraldehyde-3-phosphate dehydrogenase. Phytomedicine.

[196] Yamamoto E.S., Campos B.L.S., Jesus J.A., Laurenti M.D., Ribeiro S.P., Kallás E.G., Rafael-Fernandes M., Santos-Gomes G., Silva M.S., Sessa D.P. (2015). The effect of ursolic acid on leishmania (Leishmania) amazonensis is related to programed cell death and presents therapeutic potential in experimental cutaneous leishmaniasis. PLoS ONE.

[197] Roberto M., Junior M., Daugsch A., Moraes C.S., Queiroga C.L., Pastore G.M., Park Y.K. (2008). Comparison of volatile and polyphenolic compounds in Brazilian green propolis and its botanical origin Baccharis dracunculifolia. Cienc. Tecnol. Aliment..

[198] Mohtar L.G., Rodríguez S.A., Nazareno M.A. (2018). Comparative analysis of volatile compound profiles of propolis from different provenances. J. Sci. Food Agric..

[199] Bankova V., Popova M., Trusheva B. (2014). Propolis volatile compounds: Chemical diversity and biological activity: A review. Chem. Cent. J..

[200] Camargos H.S., Moreira R.A., Mendanha S.A., Fernandes K.S., Dorta M.L., Alonso A. (2014). Terpenes increase the lipid dynamics in the Leishmania plasma membrane at concentrations similar to their IC50 values. PLoS ONE.

[201] Moura I.C., Wunderlich G., Uhrig M.L., Couto A.S., Peres V.J., Katzin A.M., Kimura E.A. (2001). Limonene arrests parasite development and inhibits isoprenylation of proteins in Plasmodium falciparum. Antimicrob. Agents Chemother..

[202] Rosa M.D.S.S., Mendonça-Filho R.R., Bizzo H.R., Rodrigues I.D.A., Soares R.M.A., Souto-Padrón T., Alviano C.S., Lopes A.H.C.S. (2003). Antileishmanial activity of a linalool-rich essential oil from Croton cajucara. Antimicrob. Agents Chemother..

[203] Silveira M.A.D., De Jong D., Berretta A.A., dos Santos Galvão E.B., Ribeiro J.C., Cerqueira-Silva T., Amorim T.C., da Conceição L.F.M.R., Gomes M.M.D., Teixeira M.B. (2021). Efficacy of Brazilian Green Propolis (EPP-AF^®^) as an adjunct treatment for hospitalized COVID-19 patients: A randomized, controlled clinical trial. Biomed. Pharmacother..

[204] Esposito C., Garzarella E.U., Bocchino B., D’Avino M., Caruso G., Buonomo A.R., Sacchi R., Galeotti F., Tenore G.C., Zaccaria V. (2021). A standardized polyphenol mixture extracted from poplar-type propolis for remission of symptoms of uncomplicated upper respiratory tract infection (URTI): A monocentric, randomized, double-blind, placebo-controlled clinical trial. Phytomedicine.

[205] Cohen H.A., Varsano I., Kahan E., Sarrell E.M., Uziel Y. (2004). Effectiveness of an herbal preparation containing echinacea, propolis, and vitamin C in preventing respiratory tract infections in children. Arch. Pediatr. Adolesc. Med..

[206] Marchisio P., Esposito S., Bianchini S., Desantis C., Galeone C., Nazzari E., Pignataro L., Principi N. (2010). Effectiveness of a propolis and zinc solution in preventing acute otitis media in children with a history of recurrent acute otitis media. Int. J. Immunopathol. Pharmacol..

[207] Vekic J., Ivanisevic J., Zeljkovic A., Spasojevic-Kalimanovska V., Bogavac-Stanojevic N., Mihajlovic M., Janac J., Vujcic S., Miljkovic M., Zujovic D. (2020). Effect of propolis and N-acetylcysteine supplementation on lipoprotein subclasses distribution and paraoxonase 1 activity in subjects with acute respiratory infection. J. Med. Biochem..

[208] Kosari M., Noureddini M., Khamechi S.P., Najafi A., Ghaderi A., Sehat M., Banafshe H.R. (2021). The effect of propolis plus Hyoscyamus niger L. methanolic extract on clinical symptoms in patients with acute respiratory syndrome suspected to COVID-19: A clinical trial. Phyther. Res..

